# Supplementary material for: Associations of the built environment with type 2 diabetes in Asia: a systematic review
Source: BMJ Open. 2023 Apr 4;13(4):e065431. doi: 10.1136/bmjopen-2022-065431 (PMC10083821; doi:10.1136/bmjopen-2022-065431)
Supplement: Supplementary data [file bmjopen-2022-065431supp001.pdf]

BMJ Publishing Group Limited (BMJ) disclaims all liability and responsibility arising from any reliance placed on this supplemental material which has been supplied by the author(s)

Online Supplement-1

Systematic literature search strategy in Medline, Embase and Global Health using OVID platform

Concepts	No.	Term
Built Environment	1	(environment$ adj1 (design$ or green$ or pollut$ or built or food or natur$ or determinant$ or influence or support$ or approach$ or variable$ or attribute$ or barrier$ or local$ or rural$ or urban$ or object$ or perceiv$ or measure$ or obes$ or residen$ characteris$ or correlate$ or soci$ or physical$)).mp.
	2	(travel$ adj1 (active$ or passive$)).tw.
	3	(plan$ adj1 (urban$ or town$ or city$)).tw.
	4	(park$ adj1 (access$ or urban$)).tw.
	5	(space$ adj1 (green$ or open$ or natur$)).tw.
	6	(fast food$ adj1 (dens$ or restaurant$)).tw.
	7	(food$ adj1 (outlet$ or suppl$)).tw.
	8	(facilit$ adj1 (recreation$ or sport$)).tw.
	9	(pollut$ adj1 (air$ or noise$)).tw.
	10	(residen$ adj1 (loca$ or proximit$)).tw.
	11	retail densit*.tw.
	12	walkability.mp.
	13	contextual research.tw.
	14	sprawl$.ab.
	15	zoning.ab.
	16	worksite*.ab.
	17	cyclability.ab.
	18	sidewalk$.ab.
	19	crowd$.ab.
	20	pedestrian$.ab.
	21	cyclepath$.ab.
	22	aesthetic*.ab.
	23	remote$.ab.

	24	neighbo?rhood*.mp.
	25	2 or 3 or 4 or 5 or 6 or 7 or 8 or 9 or 10 or 11 or 12 or 13 or 14 or 15 or 16 or 17 or 18 or 19 or 20 or 21 or 22 or 23 or 24
Type 2 T2D mellitus	26	hyperglyc$.mp.
	27	T2D.ab.
	28	glyc?emi$.mp.
	29	(glucose adj1 (blood or high or low or $tolerance)).tw.
	30	insulin resistan$.tw.
	31	hba1$.mp.
	32	hyp?glyc$.mp.
	33	26 or 27 or 28 or 29 or 30 or 31 or 32
Observational studies	34	epidem$.mp.
	35	disease association.tw.
	36	inciden$.mp.
	37	prevalen$.mp.
	38	34 or 35 or 36 or 37
Asia	39	asia$.mp.
	40	(Afghanistan$ or Armenia$ or Azerbaijan or Bahrain or Bangladesh$ or Bhutan$ or Brunei$ or Cambodia$ or Chin$ or Cyprus or Georgia$ or India$ or Indonesia$ or Iran$ or Iraq$ or Israel$ or Japan$ or Jordan or Kazakhstan or Kuwait or Kyrgyzstan or Laos or Leban$ or Malaysia$ or Maldives or Mongolia$ or Myanmar or Burm$ or Nepal$ or North Korea$ or Oman or Pakistan$ or Palestin$ or Philippines or Qatar$ or Russia$ or Saudi Arabia$ or Singapor$ or South Korea$ or Sri Lanka$ or Syria$ or Taiwan or Tajikistan or Thailand or Timor-Leste or Turk$ or Turkmenistan or United Arab Emirates or Uzbekistan or Vietnam$ or Yemen).mp.
	41	39 or 40
Complete Search	42	1 and 25 and 33 and 38 and 41
