# Supplementary material for: Associations of the built environment with type 2 diabetes in Asia: a systematic review
Source: BMJ Open. 2023 Apr 4;13(4):e065431. doi: 10.1136/bmjopen-2022-065431 (PMC10083821; doi:10.1136/bmjopen-2022-065431)
Supplement: Supplementary data [file bmjopen-2022-065431supp002.pdf]

Online Supplement-2

Key to Quality Assessment of the selected studies

Criteria	Items	Description	Maximum Score
**Selection**	**Representativeness of the sample**	The studies choose the samples which were truly or somewhat representative of the average in the target population or not.	1
	**Study size**	The sample size the study selected was justified and satisfactory or not	2
	**Ascertainment of the exposure**	If the study applied validated measurement tool to ascertain the risk factors, we assigned two stars. Additionally, we still assigned one star to the study applied non-validated measurement tool which was available or described.	2
**Comparability**	**The Subjects in Different Outcome Groups are Comparable**	The study controlled for the confounding factors or not	1
**Outcome measures and analysis**	**Assessment of the outcome**	The study applied independent blind assessment, record linkage, self-report or no description. If it used independent blind assessment or record linkage, we assigned two stars. If it used self-report, we assigned one star.	2
	**Statistical test**	If the statistical test used to analyze the data was clearly described and appropriate, and the measurement of the association was presented, including confidence intervals and the probability level (i.e., p value), we assigned one star.	1

Based on standard reference Newcastle-Ottawa Quality Assessment scale(15)

Aarthi GR, *et al. BMJ Open* 2023; 13:e065431. doi: 10.1136/bmjopen-2022-065431

Quality score for eligible studies

S.No	Source	Selection			Comparability	Outcome measures and analysis		Total (9)	Quality
		Representativeness of the sample	Study size	Ascertainment of the exposure	The subjects in different outcome groups are comparable	Assessment of the outcome	Statistical test		
1.	Chuang et al 2011(34)	1	2	2	1	2	0	8	Very good
2.	Tong et al 2015 (45)	1	2	2	1	2	1	9	Very good
3.	Lee et al 2015 (30)	1	2	2	1	2	0	8	Very good
4.	Chen et al 2016 (43)	1	2	2	1	2	1	9	Very good
5.	Liu Fei et al 2016 (35)	1	2	2	1	2	0	8	Very good
6.	Zhang et al 2017 (22)	1	2	1	1	2	0	7	Good
7.	Fujiwara et al 2017(29)	1	2	2	1	2	0	8	Very good
8.	Biswas and Kabir 2017 (32)	1	2	2	1	2	1	9	Very good
9.	Khafaie et al 2017 (41)	1	2	2	1	2	0	8	Very good
10.	Sohn and Oh 2017(42)	1	2	2	1	2	1	9	Very good
11.	Qiu et al 2018 (39)	1	2	2	1	2	1	9	Very good
12.	Liu et al 2019 (47)	1	2	2	1	2	1	9	Very good

13.	Liang et al 2019 (36)	1	2	2	1	2	0	8	Very good
14.	Curto et al 2019 (23)	1	2	2	1	2	0	8	Very good
15.	Han et al 2019 (40)	1	1	2	1	2	1	8	Very good
16.	Lin et al 2019(26)	1	2	2	1	2	1	9	Very good
17.	Yang et al 2019 (25)	1	2	2	1	1	1	8	Very good
18.	Jacob et al 2019 (33)	0	1	2	0	2	0	5	Satisfactory
19.	Li et al 2019 (31)	1	1	2	1	2	1	9	Very good
20.	Tao et al 2019 (37)	1	2	2	1	2	1	9	Very good
21.	Yang et al 2019(27)	1	2	2	1	2	1	9	Very good
22.	Leng et al 2020 (28)	1	2	1	1	2	1	8	Very good
23.	Wong et al 2020 (46)	1	2	2	1	2	1	9	Very good
24.	Wang et al 2020 (24)	1	2	2	1	2	1	9	Very good
25.	Mehdinia et al 2020 (38)	1	2	2	1	2	1	9	Very good
26.	Shan et al 2020 (44)	1	2	1	1	2	1	8	Good

Aarthi GR, *et al. BMJ Open* 2023; 13:e065431. doi: 10.1136/bmjopen-2022-065431
