# Supplementary material for: Associations of the built environment with type 2 diabetes in Asia: a systematic review
Source: BMJ Open. 2023 Apr 4;13(4):e065431. doi: 10.1136/bmjopen-2022-065431 (PMC10083821; doi:10.1136/bmjopen-2022-065431)
Supplement: Supplementary data [file bmjopen-2022-065431supp003.pdf]

Online Supplement -3

Summary of study characteristics of articles

**Author, year & country**	**Study design**	**Sample size & Age (in years)**	**Outcome type**	**Exposure category**	**Exposure assessment**	**Confounders**	**Statistical method**	**Study Findings**
Chuang et al, 2011, Taiwan (41)	Cross-sectional	1023 participants aged ≥ 54 years	T2D risk	Air pollution	Secondary	Age, sex, body mass index, current smoking, drinking, and smooth functions of visit date and yearly temperature	Linear additive models	There was a stronger association between T2D and PM2.5 than with two gases, O3 and NO2.
Tong et al, 2015, China(53)	Cross-sectional	1430 participants from all age group	T2D risk	Air pollution	Secondary	Seasonality, temperature, humidity	Log linear generalized model	Increase in 2-day average concentrations of particulate matters like (PM10), SO2, and NO2 correspond to increases in T2D morbidity. The association was in expected direction
Lee et al, 2015, South Korea(36)	Cross-sectional	16178 participants aged ≥20 years	T2D prevalence	Walkability	Secondary	Age, sex, smoking status, drinking status and income level	Multiple logistic regression analysis	T2D mellitus is associated with walkable environments, and physical activity may contribute to the association.

Chen et al, 2016, China(51)	Cohort	27685 participants aged between18-98 years	T2D prevalence	Air pollution	Secondary	Age, sex, blood pressure (systolic/diastolic blood pressure),bmi, diabetes, treatment of diabetes, exercise activity, walking, smoking, alcohol drinking, health examination hospital, seasonality, temperature and relative humidity	Longitudinal analysis	In women, elderly and overweight people, air pollutants had a higher effect on FBG than in males, young and underweight people. This suggests that air pollution may increase the levels of FBG.
Liu et al, 2016, China(42)	Cross-sectional	11847 participants aged ≥45 years	T2D prevalence and risk	Air pollution	GIS	Location of residence (urban or rural), age, sex, educational level (low, medium, high), body mass index, smoking status (current smoker, ex- smoker and non-smoker), pack years for current smokers, drinking (>1/month, <1/month, never), type of heating energy: central heating, clean, unclean, other; type of cooking energy: clean, unclean, other) and ozone (estimated by the gbd project)	Multivariable logistic regression	Significant increases in T2D prevalence, fasting glucose levels, and hba1c levels were associated with long-term exposure to PM2.5.

Zhang et al, 2017, China (28)	Cross-sectional	2745 participants aged ≥18 years	T2D Prevalence	Accessibility to services	Secondary	County- level socioecono mic factors (e.g., natural logarithms of the local gdp per capita and the local averaged wage), health facility factors (e.g.,hospital density and health center density), demographi c features (e.g., household size, regional average age and county-level gender divisions) and other health outcomes probably influencing diabetes (e.g., obesity and hypertensio n)	Ordinary least squares, robust regression and a set of binary choice model	China's T2D prevalence is inextricably linked to the built environment as well as individual economic factors. When combined with other influential factors, China's strong economic growth has made obesity and T2D more common.
Fujiwara et al, 2017, Japan (35)	Cross-sectional	8904 participants aged ≥ 65 years	T2D Prevalence	Food environment and walkability	GIS	Each hilly assessment, perception of access to grocery stores (unit: iqr), number of parks (unit: iqr), number of hospitals (unit: iqr), population density (tertile), and land value (unit: iqr), sex, marital status, household number, income, working status, drinking, smoking, vegetable consumption, walking, going-out behavior, frequency of meeting, bmi (category), and depression	Multilevel logistic regression	There was no association between hilly neighbourhoods and T2D mellitus, but they protected against poorly managed T2D mellitus.

Biswas and Kabir, 2017, Bangladesh (39)	Cross-sectional	7544 participants aged ≥35 years	T2D Prevalence	Availability and accessibility to services	Secondary		Generalized linear mixed models	It was found that T2D was not significantly associated with distance from a health facility.
Khafaie et al, 2017, India(49)	Cross-sectional	1213 participants aged ≥46 years	T2D risk	Air pollution	Atmospheric dispersion model	Age, gender, duration of diabetes, WHR, smoking and season of enrolment	Multiple linear regression	Exposure to PM10 long-term correlates with high insulin resistance and high glycaemia.
Sohn and Oh, 2017, South Korea(50)	Cross-sectional	43941 participants with median age 46.7	T2D prevalence	Air pollution	Secondary	Age, body mass index, economic activity status, educational attainment, and smoking behavior	Multivariable logistic regression	In South Korea, PM10 and SO2 levels may be associated with T2D prevalence, but there appears to be a gender difference. Exposures to either PM10 or SO2 were significantly related to the prevalence of DM 2 among women, but not among men.
Qiu et al, 2018, China(46)	Cohort	53905 participants aged ≥65 years	T2D incidence and prevalence	Air pollution	GIS	Age, sex, calender year of entry, bmi, smoking status, alcohol drinking, physical exercise, education, monthly expenses, medication taken, self reported comorbidity including hypertension, heart disease, COPD/asthma, or cerebrovascular accident at baseline, small area characteristics (% 65+ years, % with tertiary education and income ≥ US$ 1923/month) and district	Cox regression	In Hong Kong's elderly population, long-term exposure to high levels of PM2.5 may increase the prevalence and incidence of type 2 T2D mellitus.

						smoking rate.		
Liu et al, 2019, China(55)	Cohort	39191 participants aged between 18-79 years	T2D prevalence and risk	Air pollution	GIS	Age, sex, education level, marital status, average monthly income, smoking, drinking, high fat diet, fruit and vegetable intake, physical activity, family history of diabetes, bmi	Linear regression model	There is a positive correlation between higher exposures of air pollutants and increased odds of developing type 2 T2D, along with higher fasting blood sugar levels among Chinese rural residents.

Liang et al, 2019, China(43)	Cohort	88397 participants with mean age 51.7	T2D incidence	Air pollution	GIS	Age, gender, temperature, RH, BMI, smoking status, educational level, work- related physical activity level, family history of diabetes and hypertension status, neighborhood SES including urbanicity (urban or rural) and county-level averaged years of education	Cox proportional hazard regression	There is evidence that prolonged exposure to PM2.5 is associated with T2D incidence in China. If air quality is improved in China, it will impact the reduction of T2D incidence in China.
Curto et al, 2019, India (29)	Cross-sectional	5065 participants aged ≥18 years	T2D Prevalence	Air pollution	Gravimetric sampler	Pm2.5/bc residual + age + sex + mean pm2.5/bc village + (1| village/ household) + sugar and sweets intake + physical activity + education + alcohol intake + smoking + environmental tobacco smoke + standard of living index + cooking fuel + body mass index + waist-to-hip-ratio + physician- diagnosed hypertension	Logistic mixed regression models	Women's blood glucose levels were negatively influenced by PM2.5 and black carbon (BC). In men, associations were negative for PM2.5 and positive for BC.
Han et al, 2019, China(47)	Case-control	120 participants in each group aged between 50-65	T2D prevalence	Air pollution	Particulate sampler	Age, sex, BMI, smoking status, medication usage, disease history, day of the week (DOW), temperature and RH	Linear mixed effect model	People with T2D are more susceptible to the acute cardio metabolic effects of air pollution than healthy people.

		years						
Lin et al, 2019, Taiwan (32)	Cross-sectional	341211773602 participants aged ≥ 18 years	T2D risk	Urban green space	GIS	-	Linear mixed-effect model with an individual-level random effect	Residential greenness appears to be associated with lower glucose levels. Physical activity is a possible mediator of this association.
Yang et al, 2018, China(31)	Cross-sectional	15477 participants aged ≥ 50 years	T2D prevalence	Air pollution	Secondary	Age, sex, body-mass index, education, family income, smoking, alcohol consumption, exercise, low calorie and low-fat controlled diet, sugar-sweetened soft drink consumption, family history of diabetes, and district	Linear regression model	T2D and PM2.5 are statistically significantly associated. In stratified analyses by fruit consumption, a lower association was found among participants.
Jacob et al, 2019, India (40)	Cross-sectional	410 participants aged ≥18 years	T2D prevalence	Air pollution	Gravimetric sampler		Multivariable logistic regression	People living in areas with high particulate matter exposure were 77.5% more likely to have T2D than those living in areas with less exposure.

Li et al, 2019, India(37)	Cross-sectional	5764 participants with median age 28.8	T2D risk	Food environment	GIS	Age, sex, education, occupation, standard of living index, tobacco, alcohol, and physical activity + densities or distances of highly processed/take-away food vendors	Three-level mixed-effects linear regression	Having fewer fruit and vegetable vendors and having more take-out and highly processed foods was associated with higher cardiovascular risk profiles.
Tao et al, 2019, China(44)	Cross-sectional	6627 participants aged ≥18 years	T2D risk	Air pollution	GIS	Sex, age, race, education, occupation, household income, marital status, type of home cooking fuel, family history of diabetes mellitus, smoking, passive smoking, alcohol consumption, BMI, weight change in the past year, blood lipid control, blood pressure control, season of investigation, physical activity, weekly consumption of grains, vegetables, fruit, meat products and Pm1 exposure	Multinomial logistic regression	PM2.5 and PM1 were both linked to an increased risk of impaired fasting glucose (IFG) and T2D.

Yang et al., 2019, China(33)	Cross-sectional	15,477 participants aged 18-74 years	T2D Prevalence	Urban green space	GIS	Age (years), sex (male vs. female), ethnicity (Han vs. other), household annual income (≤5000 Yuan, 5001-10,000 Yuan, 10,001-30,000 Yuan, ≥ 30,000 Yuan), and highest educational attainment (no school, primary school, middle school, junior college or higher). Air pollution, physical activity and BMI were also selected as candidate mediators	Linear regression model	Higher residential greenness appears to be beneficially associated with both T2D prevalence and glucose-homeostasis markers in Chinese adults.
Leng et al, 2020, China(34)	Cross-sectional	4155 participants aged ≥20 years	T2D prevalence	Urban green space	Secondary	Age, gender, education	Logistic regression	Green space characteristics of a neighborhood were not associated with T2D.
Wong et al, 2020, Malaysia (54)	Cross-sectional	56710 participants from all age group	T2D risk	Air pollution	Secondary	Age, gender, ethnicity, urban/rural areas, body mass index (BMI) and physical activity	Multiple logistic and linear regression model	In Malaysia, long-term exposure to O3 is a significant factor associated with under diagnosed DM. The risk of DM may be influenced by the presence of PM10, NO2 and NOx, and their roles should be explored in further detail with other interaction models.

Wang et al, 2020, China(30)	Cohort	19884 participants aged between 20-79	T2D incidence	Air pollution	Secondary	Age, gender, pollution monitoring stations, marital status, educational level, family income, work type, smoking and drinking status, physical exercise, family history of diabetes, BMI, dietary salt, oil, sugar intake, average temperature, relative humidity, the season at baseline, hypertension status, TC, TG, LDLC and HDLC	Cox proportional hazard regression	T2D incidence increased by 17% per 10μg/m3 increase in environmental PM10, and the risk rises gradually with higher PM10 concentrations. The risk of T2D incidence associated with ambient PM10 was higher among females, young to middle-aged people, overweight and obese subjects, and those with a FPG level at baseline less than 5.6 mmol/L.
Mehdinia et al, 2020, Iran(45)	Cross-sectional	482833 participants aged between 30-59 years	T2D prevalence	Air pollution	Secondary		Linear regression model	T2D prevalence and PM2.5 concentrations show a significant association.
Shan et al, 2020, China(52)	Cohort	38529 participants with mean age 44.1	T2D incidence	Air pollution	Secondary	Sex, age, smoking status, education level, personal income, marital status, occupational exposure, exercise, fruit consumption, vegetable consumption, seafood consumption, meat consumption, poultry consumption and alcohol consumption	Cox proportional hazard regression	Among Chinese adults, it was found that long-term exposure to high levels of PM10, SO2, and NO2 increased risk of T2DM incidence and mortality.

Kusuma et al, 2022, South Asia(38)	Cross sectional	12167 participants with median age 45.5	T2D prevalance	Food environment	GIS	Family history of diabetes, BMI, dietary salt, oil, sugar intake, average temperature, relative humidity, the season at baseline, hypertension status, TC, TG, LDLC and HDLC	Ordinary least squares multivariate regression	An increase in blood glucose levels and the likelihood of being diagnosed with diabetes is associated with having at least one FFR locally by 16% and 19% respectively. FFR density had a stronger positive association with blood glucose levels among women than men. In contrast, FFR proximity had a stronger association with blood glucose levels among men and those with higher incomes.
Liu et al, 2023, China(48)	Cohort	19121 participants aged ≥45 years	T2D risk	Air pollution	Secondary	Demographic characteristics, indoor pollution, life behaviours and ambient temperature	Cox proportional hazards regression model	The long-term exposure to PM2.5 can increase the risk of type 2 diabetes and make them more prone to arthritic problems.
