# Supplementary material for: Associations of the built environment with type 2 diabetes in Asia: a systematic review
Source: BMJ Open. 2023 Apr 4;13(4):e065431. doi: 10.1136/bmjopen-2022-065431 (PMC10083821; doi:10.1136/bmjopen-2022-065431)
Supplement: Author's manuscript [file bmjopen-2022-065431.draft_revisions.pdf]

BMJ Open is committed to open peer review. As part of this commitment we make the peer review history of every article we publish publicly available.

When an article is published we post the peer reviewers' comments and the authors' responses online. We also post the versions of the paper that were used during peer review. These are the versions that the peer review comments apply to.

The versions of the paper that follow are the versions that were submitted during the peer review process. They are not the versions of record or the final published versions. They should not be cited or distributed as the published version of this manuscript.

BMJ Open is an open access journal and the full, final, typeset and author-corrected version of record of the manuscript is available on our site with no access controls, subscription charges or pay-per-view fees (http://bmjopen.bmj.com).

If you have any questions on BMJ Open's open peer review process please email info.bmjopen@bmj.com

BMJ Open

# Built Environment and Type 2 Diabetes in Asia: A Systematic Review and Meta-Analysis

| | |
|---|---|
| Journal: | *BMJ Open* |
| Manuscript ID | bmjopen-2022-065431 |
| Article Type: | Original research |
| Date Submitted by the Author: | 07-Jun-2022 |
| Complete List of Authors: | Raveendiran, Aarthi; Madras Diabetes Research Foundation, Research Operations; SRM Institute of Science and Technology<br>Mehreen, T S; Imperial College London, School of Public Health; Madras Diabetes Research Foundation, Research Operations<br>Moosawi, Suzana; Imperial College London Faculty of Medicine, Epidemiology<br>Kusuma, Dian; Imperial College Business School, Centre for Health Economics and Policy Innovations<br>Harish, Ranjani; Madras Diabetes Research Foundation, Translational Research<br>Pradeepa, Rajendra; Madras Diabetes Research Foundation<br>Venkatasubramanian, Padma; SRM Institute of Science and Technology<br>Mohan, V; Madras Diabetes Research Foundation<br>Anjana, Ranjit Mohan; Madras Diabetes Research Foundation; SRM Institute of Science and Technology<br>Fecht , D ; Imperial College London, MRC Centre for Environment and Health, School of Public Health |
| Keywords: | General diabetes < DIABETES & ENDOCRINOLOGY, EPIDEMIOLOGY, Health & safety < HEALTH SERVICES ADMINISTRATION & MANAGEMENT, PREVENTIVE MEDICINE, PUBLIC HEALTH |
| | |

SCHOLARONE™
Manuscripts

*I, the Submitting Author has the right to grant and does grant on behalf of all authors of the Work (as defined in the below author licence), an exclusive licence and/or a non-exclusive licence for contributions from authors who are: i) UK Crown employees; ii) where BMJ has agreed a CC-BY licence shall apply, and/or iii) in accordance with the terms applicable for US Federal Government officers or employees acting as part of their official duties; on a worldwide, perpetual, irrevocable, royalty-free basis to BMJ Publishing Group Ltd ("BMJ") its licensees and where the relevant Journal is co-owned by BMJ to the co-owners of the Journal, to publish the Work in this journal and any other BMJ products and to exploit all rights, as set out in our licence.*

*The Submitting Author accepts and understands that any supply made under these terms is made by BMJ to the Submitting Author unless you are acting as an employee on behalf of your employer or a postgraduate student of an affiliated institution which is paying any applicable article publishing charge ("APC") for Open Access articles. Where the Submitting Author wishes to make the Work available on an Open Access basis (and intends to pay the relevant APC), the terms of reuse of such Open Access shall be governed by a Creative Commons licence – details of these licences and which Creative Commons licence will apply to this Work are set out in our licence referred to above.*

*Other than as permitted in any relevant BMJ Author's Self Archiving Policies, I confirm this Work has not been accepted for publication elsewhere, is not being considered for publication elsewhere and does not duplicate material already published. I confirm all authors consent to publication of this Work and authorise the granting of this licence.*

For peer review only

# Built Environment and Type 2 Diabetes in Asia: A Systematic Review and Meta-Analysis

Garudam Raveendiran Aarthi [1,4,5], Thaharullah Shah Mehreen [1,5], Suzana Al Moosawi[5], Dian Kusuma[6], HarishRanjani[3], Rajendra Pradeepa [1], Venkatasubramanian Padma[4], Viswanathan Mohan[2], Ranjit Mohan Anjana[2,4], Daniela Fecht[7,8]

1.Department of Research Operations, Madras Diabetes Research Foundation, Chennai, India

2. Department of Diabetology, Madras Diabetes Research Foundation, Chennai, India

3. Department of Translational Research, Madras Diabetes Research Foundation, Chennai, India

4.School of Public Health, SRM Institute of Science and Technology, Kattankulathur, India

5.School of Public Health, Imperial College London, London, UK

6.Centre for Health Economics & Policy Innovation, Imperial College Business School, London, UK

7.MRC Centre for Environment and Health, Department of Epidemiology and Biostatistics, School of Public Health, ImperialCollege London, London, UK

8. National Institute for Health Research Health Protection Research Unit on Chemical and Radiation Threats and Hazards, Department of Epidemiology and Biostatistics, School of Public Health, Imperial College London, London, UK

**ADDRESS FOR CORRESPONDENCE**

**Dr. Daniela Fecht**
MRC Centre for Environment and Health,
School of Public Health,
Imperial College London,
Norfolk Place
London W2 1PG, UK
Email: d.fecht@imperial.ac.uk

**Dr. Ranjit Mohan Anjana**
Madras Diabetes Research Foundation,
4, Conran Smith Road, Gopalapuram,
Chennai- 600086, INDIA
Phone: 91-44-4396 8888
Fax: 91-44-28350935
Email: dranjana@drmohans.com



For peer review only - http://bmjopen.bmj.com/site/about/guidelines.xhtml

For peer review only

## ABSTRACT

**Objectives:** Our study aimed to systematically review and syntheses findings on the impact of built environment characteristics on type 2 diabetes (T2D) in Asia.

**Design:** Systematic review of the literature

**Setting:** We focus in our review on Asian cities.

**Participants:** General population aged 18 years and above living in Asia.

**Results** Five broad categories of built environment characteristics were associated with T2D in Asia. These included urban green space, walkability, food environment, availability and accessibility of services such as recreational and healthcare facilities and air pollution. We found very strong evidence of associations of particulate matter ($PM_{2.5}$, $PM_{10}$), nitrogen dioxide and sulfur dioxide ($p$<0.001) with T2D risk.

**Conclusion** Several built environment attributes were significantly related to T2D in Asia. When compared to western countries, very few studies have been conducted in Asia. Further research is, therefore, warranted to establish the importance of the built environment characteristics on T2D. Such evidence is essential for public health and planning policies to (re)design neighborhoods that help improve public health across Asian countries.

**Registration:** This systematic review was conducted according to PRISMA guidelines and registered with PROSPERO database (CRD42020214852).


**Keywords:** diabetes, epidemiology, health and safety, preventive medicine, public health



For peer review only - http://bmjopen.bmj.com/site/about/guidelines.xhtml

**Strengths and Limitations**

- This is the first systematic review to synthesize the evidence of the impact of built environmental characteristics including urban green space, walkability, food environments, availability and accessibility to services (recreational and healthcare), and air pollution on T2D in Asia.
- Due to the heterogeneity in built environment characteristics, outcome measures and methodology used in the eligible studies, a formal meta-analysis was not feasible.
- The weighted *z*-test was used to quantify the strength of evidence and syntheses the findings, but only selected air pollutants were assessed due to lack of studies on other categories.



For peer review only - http://bmjopen.bmj.com/site/about/guidelines.xhtml

## INTRODUCTION

Non-communicable diseases (NCDs) are a key cause of poor health globally, and account for seven out of ten deaths worldwide. In low- and middle-income countries (LMICs), NCDs are a major barrier to development (1). Type 2 diabetes (T2D) is a large contributor to the NCD burden and a major risk factor for cardiovascular disease (CVD), affecting the quality of life and reducing life expectancy by about 4 to 8 years compared to those without the disease(2). Approximately 537 million people worldwide are affected by T2D and the number is predicted to rise to 783 million by 2045(3). LMICs are expected to see an 87% increase in T2D prevalence, with the Indian subcontinent predicted to experience the largest increase(4). More than 60% of people with T2D in the world are Asians(5).

NCDs and T2D are primarily explained by genetic, demographic, and lifestyle factors; however, recent findings indicate that an increasing proportion of NCDs are attributable to environmental factors including the built environment(1, 6).The built environment encompasses the physical and built environment in which people live, work and socialize, including man-made structures such as buildings, transportation systems, other physical surroundings constructed by human and natural features such as parks and urban forests(7, 8). The built environment can have both positive and negative impacts on NCDs. Factors such as ambient air pollutants and lack of physical activity, for example can have negative impacts on health(9, 10). Key built environment characteristics which improve public health include environments which promote physical activity, healthy eating and low pollution. The growth of cities without planned infrastructure to facilitate healthy lifestyle choices constrains opportunities for improved public health(11).

With healthier environments, 23% of deaths can be prevented worldwide(12). In Western countries, there is evidence of the positive and negative impacts of the built environment on health; however, research on Asian cities is lacking to assist in future city planning and public



For peer review only - http://bmjopen.bmj.com/site/about/guidelines.xhtml

health strategies. We conducted a systematic review of the existing evidence on the relationship between built environment characteristics and T2D in Asian adults and assessed the strength and direction of the existing evidence.

## METHODS AND ANALYSIS

### Search strategy

A comprehensive systematic bibliographic search was conducted to identify epidemiological evidence on the impact of the built environment on T2D in Asian countries. The systematic review followed the Preferred Reporting Items for Systematic Reviews and Meta-Analysis (PRISMA) guidelines(13). The protocol was registered with the International Prospective Register of Systematic Reviews (PROSPERO) database (CRD42020214852). A comprehensive search strategy identified peer-reviewed journal articles from inception to 3rd February 2021 using the electronic bibliographic databases Medline, Embase and Global Health. The reference search was carried out again on newly identified articles until no more relevant articles were discovered. The search strategy included combinations of keywords related to the built environment (accessibility of services, air pollution, food environment, urban green space, walkability, density and clustering measures), health outcome (T2D), study design (observational studies including cohort, cross-sectional and case-control studies) and study area (Asia).

**Patient and Public Involvement:** There was no direct involvement of patients or the public in this study.

### Eligibility criteria

Study titles and abstracts were examined to assess their eligibility for inclusion based on predefined participant, intervention, comparison, and outcome (PICO) criteria, shown in Table1. Only studies published in the English language were considered. As diabetes



For peer review only - http://bmjopen.bmj.com/site/about/guidelines.xhtml

accounts for the majority of T2D cases in the general community(>90 %), studies that did not specify the type of diabetes were included.

**Table 1. Inclusion and exclusion criteria for literature search**

| | **Inclusion criteria** | **Exclusion criteria** |
|---|---|---|
| *Population* | Adults aged 18 years and above residing in Asia. | Animal studies and those involving specific populations such as pregnant women. |
| *Intervention* | Studies exploring any of the following built environment features:<br>• Walkability: land-use mix, residential density;<br>• Recreational facilities: green space, public open space, parks, playgrounds;<br>• Destinations: restaurants, shops;<br>• Routes: amount of slope, traffic intensity, street connectivity;<br>• Environmental pollutants: noise, air pollution. | Those that do not meet the criteria of built environment features and studies in an experimental setting. |
| *Comparators* | NA | NA |

For peer review only



For peer review only - http://bmjopen.bmj.com/site/about/guidelines.xhtml

| | | |
|---|---|---|
| *Outcome* | Studies reporting outcomes on: prevalence/incidence of T2D and pre-diabetes, fasting blood glucose (FBG) and 2-hour plasma glucose (2hPG), glycated haemoglobin (HbA1c), insulin and Homeostatic model assessment of insulin resistance (HOMA-IR) levels. | Studies that evaluated admissions to hospital or emergency departments due to T2D. |
| *Study type* | Observational studies on the association of built environment features with T2D outcomes: including cohort, cross sectional and case-control studies. | Controlled trials, reviews, case reports and interventional studies. Specific publications which do not report original scientific research including letters, editorials, interviews, and legal documents. |

For peer review only

**Screening, data extraction and preparation**

Online Supplement File 1 provides a comprehensive overview of the search terms, including MESH terms used for MEDLINE, Embase, and Global Health using OVID databases. Using Covidence, an online, systematic review tool, duplicates were removed and the identified articles were screened against the pre-defined study selection criteria, independently by two reviewers (GRA and TSM). At all screening stages, title, abstract and full text, discrepancies were resolved through discussion with a mediator (HR). A standardized data extraction form



For peer review only - http://bmjopen.bmj.com/site/about/guidelines.xhtml

was used for collection of information on methodology, outcome and exposure measures, including study characteristics (year of publication, study design, sample size, country), participant characteristics (gender, age range), health outcomes (T2D outcome and measurement method), built environment characteristics (type, measurement tool), and statistical methods. Data extraction was performed by reviewer TSM and data extraction was assessed by reviewer GRA.

**Quality assessment**

To assess the quality of included studies, the Newcastle - Ottawa Quality Assessment Scale (NOS) for Observational Cohort inan adapted version for cross-sectional Studies was used(14). The quality assessment was performed by reviewer TSM. Each study was scored for quality by blinding the authors, institutions, country, and journal. In evaluating the quality of each study, six criteria were used: (i) representativeness of the sample, (ii) study size, (iii) ascertainment of the exposure, (iv) comparable subjects in different outcome groups, (v) assessment of the outcome, (vi) statistical test. Based on these criteria, star ratings were awarded. The six criteria were categorized into three broader categories relating to: a) the study population, b) the comparability of population and c) characterization of exposure/outcome variables. The maximum number of stars awarded for the study population was 5 stars while the comparability of populations and characterization of exposure/outcome variables were awarded 1 star and 3 stars, respectively. The study quality was evaluated based on the total number of stars assigned to each study, with eight to nine stars defined as "very good" quality, six to seven stars defined as "good" quality, four to five stars defined as "satisfactory" quality, and zero to four stars defined as "unsatisfactory" quality. Online Supplement File 2 provides the summary of quality assessment using the NOS.



For peer review only - http://bmjopen.bmj.com/site/about/guidelines.xhtml

**Data synthesis**

Due to the heterogeneity in built environment characteristics, outcome measures and methodology used in the eligible studies, a formal meta-analysis was unfeasible. Heterogeneity in built environment studies is a common issue that has been described by other authors (15-17). To overcome this issue, a semi-quantitative meta-analytical method was used which has been developed and described in detail previously(18, 19). This method synthesizes studies based on a weighted *z*-test, which considers the direction of findings, either in the expected or unexpected direction, and weights their level of significance based on the *p*-value(20) as follows:

| Statistically significant | α | *z*-value |
|---|---|---|
| Yes (expected direction) | 0.05 | 1.96 |
| | 0.1 | 1.64 |
| No (null) | 0 | 0 |
| Yes (unexpected direction) | 0.1 | - 1.64 |
| | 0.05 | - 1.96 |

For peer review only

In order to calculate the *z*-score associated with a specific exposure–outcome relationship, the sum of *z*-values was divided by the square root of the sum of squared weights, using the quality assessment scores of individual studies as weights. After calculating the weighted *z* -value, the two-tailed *p*-value was used to determine whether the evidence was strong or weak using the following criteria: $p<0.05$ for weak evidence, $p<0.01$ for strong evidence, and $p<0.001$ for very strong evidence. The following equation was used to derive a summarized weighted *z* -value:



For peer review only - http://bmjopen.bmj.com/site/about/guidelines.xhtml

$$Weightedz = \frac{\sum weight_j z_j}{\sqrt{\sum weight_j^2}}$$

## RESULTS

Initially, 5180 articles were identified, of which 2105 were duplicates. After screening of articles according to eligibility criteria, 26 articles were eligible for inclusion in the systematic review (Figure 1).

Out of the 26 included studies, the majority were conducted in China (53.9%), followed by India (19.3%), South Korea (7.8%), Japan (3.8%), Taiwan (3.8%), and Bangladesh (3.8%). The earliest study was published in 2011, over 75% of studies after 2017. Cross-sectional study designs were used in 73.1% of the studies, while cohort study designs and case control were used in 23.1 % and 3.8 % of the studies, respectively. All studies included both male and female participants. Sample sizes ranged from 86 to 7,73,602 participants, and different methods for participant recruitment and data collection were used such as medical records and self-reported health status. Study sample sizes <1000 were used by 7.6%, and studies using sample sizes 1001-2500 and >2500 were used by 11.6% and 80.8%, respectively. The quality score ranged from5 to 9. The mean quality score was 8 (standard deviation 1.03) indicating overall good and very good quality. The detailed characteristics from all the studies included in the systematic review are presented in Online Supplement File3.

The majority of studies used objectively collected data on diabetes (*n*=19), with the outcome being evaluated by biomedical tests or retrieved from registry records. One study used self-reported data(21) and three studies used a combination of hospital records and self-reported data(22-24). The outcomes included diabetes and pre-diabetes prevalence and incidence (*n* = 19), levels of fasting blood glucose (FBG) (*n* = 9), 2-hour plasma glucose (2hPG) (*n* = 1), glycated hemoglobin (HbA1c) (*n* = 3), insulin level (*n* = 1), and homeostatic model



For peer review only - http://bmjopen.bmj.com/site/about/guidelines.xhtml

assessment of insulin resistance (HOMA-IR) ($n$ = 1). Outcome variables were expressed as changes in continuous measurements in longitudinal or cohort studies (for example, changes in FBG levels from baseline to follow-up) or as T2D prevalence reported in cross sectional studies.

Studies explored a wide range of built environment characteristics which we categorized into i) urban green space($n$ = 3), ii) walkability($n$ = 2),iii) food environment($n$ = 2), iv) availability and accessibility to services($n$ = 2), and v) air pollution($n$ =18). Studies used either objectively measured exposure derived using geographic information systems (GIS)($n$=12) or audit measures($n$=12). The sphere of influence of exposures was defined as either a circular buffer($n$=2) or street-network buffer($n$=1) around the participants' place of residence, ranging from 400meters to 1600meters. Some studies also used proximity to destinations($n$=2) and modeled air pollution concentration at place of residences ($n$=7).

**Built environment and T2D**

The results are presented for each built environment category separately. Study characteristics and quality assessment for all included studies are presented in Online Supplement File 2 and 3, respectively. Due to fewer than 5studies on most built environment categories, the weighted $z$- score was calculated only for air pollution.

**Urban green space**

The term urban green space is broadly used to describe a range of greenareas including parks, ground cover vegetation, street trees, and green roofs. Access and availability of urban green space is hypothesized to reduce T2D. Three studies investigated the association of urban green space with T2D(25-27), two studies conducted in China(26, 27) and one in Taiwan(25). All studies used FBG as a measure of T2D; one study used 2-h glucose, HOMA-IR(26). Greenness was assessed with Normalized Difference Vegetative Index (NDVI)(25, 26), soil



For peer review only - http://bmjopen.bmj.com/site/about/guidelines.xhtml

adjusted vegetation index (SAVI)(26), while other components of green space were assessed with green space ratio, green vision index, and evergreen tree configuration(27). The statistical analysis of the data was performed using logistic regression(25), binary logistic regression model(26) and linear mixed effect models(25, 27). Greenness was associated with lower glucose levels in Taiwan(24) and China(26)($p<0.05$). However, the other exposure variables used to measure urban green space did not show an association with T2D(25).

**Walkability**

Higher walkability is hypothesized to encourage people to walkin their local community, increase physical activity and is consequently hypothesized to have a positive effect on T2D.Two studies assessed walkability using a cross-sectional design in Japan(28) and South Korea(29). Both studies assessed T2D using FBG levels along with this one study used HbA1c, and medical history, both studies recruiting study participants from secondary data(28). The study conducted in Japan used the availability of parks, slope, and land use mix to measure walkability, while the study from South Korea used street patterns, slopes, and neighborhood amenities, such as the amount of neighborhood park space, number of shopping malls, and distance between communities and malls, which were both quantified and qualitatively assessed through site observations and GIS analyses. Both studies assessed T2D based on FBG levels; one study assessed HbA1c levels(28). Multilevel regression model(28) and multiple logistic regression analysis(29) were used to perform the statistical analysis. According to the study conducted in South Korea, people who lived in communities with better street networks, defined as grid layout of pedestrian pathways, and pleasant landscape, including street trees, had a lower risk of T2D(odds ratio (OR):0.83, 95% confidence intervals (CI):0.77 to0.91). Other associations of exposure variables with T2D



For peer review only - http://bmjopen.bmj.com/site/about/guidelines.xhtml

were not statistically significant. The study from Japan, did not find a statistically significant impact of a hilly environment on T2D. Steeper slopes, on the other hand, reduced the risk of poorly controlled T2D (HbA1c levels of ≥7.5% in healthy people without any co-morbidity or ≥8% in the elderly).

**Food environment**

The food environment facilitates either health or unhealthy food acquisition and consumption within the wider food system and can therefore have both positive and negative effects on T2D. The food environment was examined by two studies(28, 30). Among participants aged 65 and older, one study examined the perception of access to grocery stores for fresh fruits and vegetables in Japan(28). The other study, conducted in India, collected information on shops and services selling food, tobacco, and alcohol, as well as the density of vendors. Both studies assessed T2D based on FBG, HbA1c and insulin levels. The study from Japan, found no significant association between perception of access to grocery stores and T2D. Using three levels of mixed-effect linear regression, the density and distance of fruit and vegetable vendors and highly processed/takeout food vendors were analyzed in relation to T2D in the Indian study. The associations between highly processed and take-away food vendor density and FBG (OR: 0.30, 95%CI: -0.14 to 0.74) and insulin (OR: 0.13, 95%CI: -0.04 to 0.29) were statistically non-significant(30). There was no association between distance to the nearest fruit and vegetable vendor or highly processed and take-away food vendor and FBG or insulin levels(28).

**Availability and accessibility to services**

Two studies conducted in China(21) and Bangladesh(31) examined the relationship between the availability and accessibility of services like healthcare, recreational facilities andT2D.



For peer review only - http://bmjopen.bmj.com/site/about/guidelines.xhtml

Both studies used a cross sectional study design and collected data from secondary sources from medical records. T2D was measured using FBG and self-reportedT2D. Generalized linear mixed models(31) and ordinary least squares models(21) were used for statistical analyses. T2D was more prevalent in areas of greater distance from health facilities, such as community clinics, but the difference was statistically not significant (OR: 1.05, 95%CI: 0.6 to 1.61)(21). In the study conducted in Bangladesh, the association of distance to services with T2D was statistically non-significant except for the distance to the cinema hall (OR: 1.76, 95% CI: 1.04 to 2.98).

**Air pollution**

Air pollution has been hypothesized to increase the prevalence of T2D. Overall, 18 studies on air pollution were conducted in Asia. Particulate matter (PM) of various particle sizes, black carbon(BC), nitrogen dioxide($NO_2$), ozone($O_3$), and sulphur dioxide($SO_2$) were the main air pollutants studied. Eleven studies were cross-sectional, followed by cohort studies (*n*=6) and one case-control studies. There were 11 studies conducted in China, followed by three in India, and one each in South Korea, Iran, Taiwan, and Malaysia. 482,833 people were enrolled in a cross-sectional study with the largest sample size. Particulate matter $PM_{2.5}$ (*n*=11)(22, 24, 32-39) was found to be the most commonly examined pollutant in relation to T2D, followed by $PM_{10}$ (*n*=8)(23, 33, 40-45), $NO_2$ (*n*=6)(33, 42-46), $SO_2$ (*n*=6)(40-45), $NO_x$ (*n* = 2)(40, 45), ozone (*n* = 2) (33, 45), $PM_1$(*n*=2), (36, 46) and BC (*n*=1)(22). There were six studies that used medical history and T2D treatments to assess T2D status, with others using FBG, HbA1c, and Fasting plasma glucose, and one using random capillary blood glucose to assess T2D. Several studies used secondary data such as data fromKorea Community Health Survey data, Malaysian National Health and Morbidity Surveys for health, and local environmental monitoring centers to analyze air pollution. Other studies used satellite-based



For peer review only - http://bmjopen.bmj.com/site/about/guidelines.xhtml

289 spatial statistical models, two used spatio-temporal models, two used gravity samples, and
290 one used AERMOD dispersion models.

291 Six studies found evidence of the adverse effects of air pollutants like $PM_{2.5}$, $PM_{10}$, $NO_2$ and
292 $SO_2$ on T2D, with more severe effects among women, the elderly and vulnerable people(23,
293 35, 38, 41, 42, 44). Eleven studies evaluated $PM_{2.5}$ with ten reporting a positive association
294 between air pollution and T2D, i.e., when air pollution is increased, the risk for T2D also
295 increases, and one study reported a negative association, i.e., when air pollution is increased,
296 the risk for T2D decreases(31). The majority of studies indicate that the risk of T2D increased
297 when exposure to $PM_{10}$ ($n$=8) or $SO_2$ ($n$=6) increased. One study reported no association
298 between $PM_{10}$ and T2D in men(34). Six studies on $NO_2$ found a significant increase in T2D
299 risk when exposed to it.

300 By using the $z$- test, we synthesized the evidence for $PM_{2.5}$, $PM_{10}$, $NO_2$, and $SO_2$,
301 separately. The two-tailed $p$-value from the weighted $z$-value for $PM_{2.5}$, $PM_{10}$, $NO_2$ and $SO_2$
302 and T2D was $p<0.001$, indicating a very strong association. The number of studies for $NO_x$,
303 ozone, $PM_1$ and black carbon was too small to derive $z$ statistics.

## 304 DISCUSSION

305 This is the first systematic review to synthesize the evidence of the impact of built
306 environmental characteristics including urban green space, walkability, food environments,
307 availability and accessibility to services (recreational and healthcare), and air pollution
308 onT2D in Asia. We found strong evidence of an association of air pollutants such as $PM_{2.5}$,
309 $PM_{10}$, $NO_2$, and $SO_2$with higher incidence/risk/prevalence of T2D. Other built environment
310 categories lack consistent evidence of an association with T2D.

311 We found that measures for urban green space including green space ratio, green vision
312 index, and evergreen configuration were not significantly associated with T2D(21, 27, 31). In



For peer review only - http://bmjopen.bmj.com/site/about/guidelines.xhtml

contrast to our findings, some cross-sectional studies in Western countries found lower rates of T2D among people exposed to more green spaces, including studies from the Netherlands(47)and the United Kingdom(48). A Norwegian study, however, did not find an association between green space and T2D, which the authors argued might be due to the fact that most of the residents had easy access to green spaces(49). Similarly to results of two studies conducted in Taiwan(25) and China(26), an American cross-sectional study found an association between greenness, measured using NDVI and T2D(50).NDVI was also associated with reduced risk of T2Dincidence in a longitudinal study conducted in Canada(51). A study in the United States found associations with T2D prevalence in a cross-sectional study but not with T2D incidence in a longitudinal study(52).A systematic review conducted globally to examine the association between green space exposure as measured by NDVI and T2D supported the notion that people and communities exposed to greenness, especially in their neighborhood, have a lower risk of T2D(53). Over the past few years, South Asia's urban green space has steadily diminished. Green spaces and conservation areas are currently under threat, primarily because housing construction in cities is unplanned and disorganized(54).

Walkability is hypothesized to increase physical activity and therefore might contribute to lower T2D rates. An effective neighborhood design considers how land-use decisions affect people's access to essential services in their communities. Completing, connecting and compacting a neighbourhood has been associated with health benefits such as increased physical activity, safer and easier transportation, enhanced employment productivity, and social inclusion. Strategies to encourage connectivity include enhancing walkability, establishing mixed land use, and designing complete and compact neighborhoods(55). An Australian study indicated that hilly neighborhoods may prevent T2D(56),while the study conducted in Japan in a hilly neighborhood, showed no link between walkability and



For peer review only - http://bmjopen.bmj.com/site/about/guidelines.xhtml

T2D(28).This difference might be due to the specific population group which focused on the elderly in the Japan study. Higher built-up density and reduced greenness were found to be connected with lower T2D rates and physical inactivity in Kerala, India(57). A study in South Korea found that participants in communities that have better street networks like grid pattern of pedestrian sidewalks and attractive scenery were less likely to have T2D(29). This is in line with findings from the Australian study indicating that study participants who lived in areas with better walkability were less likely to develop T2D(58).Our systematic review found that there is currently not much research carried out on particular built environmental attributes related to accessibility to healthcare centers in Asia. The need for longitudinal studies is thus imperative to identify such specific built environment attributes that may influence T2D outcomes in order to establish future urban design regulations for healthier and more efficient communities. The effects of walking in neighborhoods can be increased by providing pedestrian paths that are usable and unobstructed, by implementing motor-traffic reduction strategies, by increasing good street connectivity, and by providing parks, green space, playgrounds and recreation areas(59).

Our systematic review found consistent evidence of an association of the food environment withT2D risk/prevalence. An increase in fruit and vegetable vendor availability was associated with a lower T2D risk in an Indian study(30). In this study, there was strong evidence for an association between T2D risk and food vendor density within 400metres of the place of residence, while the association between take-away/processed food vendor availability and T2D was not significant. Two studies conducted in America and the United Kingdom discovered a link between increased number of fast-food restaurants and convenience stores and a higher prevalence of T2D(60, 61). Four studies conducted in the United States and Australia found no significant association between supermarket/grocery store availability and T2D prevalence(61-65).Various socio-cultural and economic norms

For peer review only



For peer review only - http://bmjopen.bmj.com/site/about/guidelines.xhtml

363 influence how people purchase and consume food, which may explain differences in the
364 association between food availability and T2D risk in different countries(66).
365
366 We found consistent and strong evidence of the impact of air pollution on T2D. Higher levels
367 of $PM_{10}$, $PM_{2.5}$ were associated with an increased risk of T2D incidence and prevalence;
368 higher $NO_2$ exposure was associated with a higher T2D prevalence, but no statistically
369 significant association with $O_3$ was found. In a previous systematic review on air pollution
370 and T2D, with most studies from North America and Europe(67), results were consistent with
371 our study. Another meta-analysis showed consistent evidence of high exposure to air
372 pollution being linked with greater T2D risk/prevalence(68) but identified a lack of studies
373 from LMICs.
374
375 In this current systematic review, we identified a paucity of evidence from a majority of
376 countries in Asia. In particular, studies from Southeast Asia, including Indonesia, the fourth
377 most populous country in the world, are lacking, however, there are some ongoing studies in
378 these countries where the findings are not available yet. Only seven cohort studies were
379 conducted using a nationally representative sample while the other studies selected samples
380 from specific regions such as specific cities, towns or counties. Hence, this systematic review
381 might not be representative of the Asian population but this is the most available evidence till
382 date. Most of the studies in our review measured built environment attributes at a single point
383 in time, however, longitudinal exposure data can be beneficial to detect changes in exposure
384 in relation to changes is T2D(69).
385
386 Overall, evidence from LMICs in Asia on the relationship between the built environment and
387 T2D is still lacking. Despite the vast amount of literature exploring the traditional risk factors



For peer review only - http://bmjopen.bmj.com/site/about/guidelines.xhtml

388 for NCDs, little attention has been directed towards specific factors of the built environment
389 influencing T2D outcomes in Asian populations. Depending on the quality and characteristics
390 of the built environment, there can be large differences between developed and developing
391 countries, which can impact CVD and T2D patterns among Asian residents(70). The
392 urbanization trend in Asia is expected to undergo a significant shift in the next decade. In
393 light of this circumstance, urban planners have a crucial opportunity to design smart cities
394 and environments that encourage an healthy lifestyles among their
395 residents(71). Nevertheless, this prime target cannot be achieved without a profound evidence
396 baseof the relationship between built environment and health and wellbeing(72).
397 Understandingthe role that the built environment plays in influencing disease risk factors may
398 help prevent the onset or deterioration of T2D and CVD with consequently vast benefits for
399 public health.
400

401 **CONCLUSION**

402 There is an urgent need for more primary research on the impact of the built environment on
403 T2D in Asia. Such studies should be carefully designed to understand the role of potential
404 confounders, risk factors and effect modifiers. Our systematic review has identified
405 significant gender differences and future studies should further assess the modifying role of
406 socioeconomic status and ethnicity.

407 **Ethical Approval:** Ethical approval was not necessary because no human or animal samples were
408 used. This review used only secondary data.

409 **Contributor ship Statement:** Search strategies were developed and guided by SAM & DF,
410 and the searches were conducted independently by GRA and TSM, and conflicts were
411 resolved by HR. Upon completion of the data synthesis and manuscript, a draft of the
412 manuscript was shared with other authors for their inputs and corrections were made based on
413 their inputs. Researchers involved in this study were all acknowledged as authors.



For peer review only - http://bmjopen.bmj.com/site/about/guidelines.xhtml

**Competing Interest:** None declared.

**Funding:** This work was supported by the MRC Centre for Environment and Health, which is currently funded by the Medical Research Council (MR/S019669/1, 2019-2024). This study is part funded by the National Institute for Health Research (NIHR) Health Protection Research Unit in Chemical and Radiation Threats and Hazards, a partnership between UK Health Security Agency and Imperial College London. Infrastructure support for the Department of Epidemiology and Biostatistics was provided by the NIHR Imperial Biomedical Research Centre. The first author (GRA) worked with the ICL team as a visiting researcher in the School of Public Health as part of a training programme funded by the National Institute for Health and Care Research (NIHR's) Short Placement Award for Research Collaboration(NIHR GHR SPARC PILOT 01-19-06). The second author (TSM) was a student at ICL who pursued her M.Sc Epidemiology in the School of Public Health, entirely funded by the National Institute for Health and Care Research- Global Health Research Unit (NIHR GHRU) project. This research was also supported by the National Institute for Health Research (NIHR) (16/136/68) using UK aid from the UK Government to support global health research. The views expressed in this publication are those of the authors and not necessarily those of the NIHR,UK Health Security Agency or the UK Department of Health and Social Care.

**Data sharing statement:** The data used for this systematic review is available on online supplement file 3.

**References**

1. Forouzanfar MH, Afshin A, Alexander LT, Anderson HR, Bhutta ZA, Biryukov S, et al. Global, regional, and national comparative risk assessment of 79 behavioural, environmental and occupational, and metabolic risks or clusters of risks, 1990–2015: a systematic analysis for the Global Burden of Disease Study 2015. The Lancet. 2016;388(10053):1659-724.
2. Sarwar N, Gao P, Seshasai SR, Gobin R, Kaptoge S, Di Angelantonio E, et al. Diabetes mellitus, fasting blood glucose concentration, and risk of vascular disease: a collaborative meta-analysis of 102 prospective studies. Lancet. 2010;375(9733):2215-22.
3. Atlas D, IDF Diabetes Atlas,. Brussels, Belgium: International Diabetes Federation, 2021. 2021; 10th edn. .
4. Abate N, Chandalia M. Ethnicity, type 2 diabetes & migrant Asian Indians. Indian J Med Res. 2007;125(3):251-8.
5. Hu FB. Globalization of diabetes: the role of diet, lifestyle, and genes. Diabetes Care. 2011;34(6):1249-57.
6. Landrigan PJ, Fuller R, Acosta NJR, Adeyi O, Arnold R, Basu NN, et al. The Lancet Commission on pollution and health. Lancet. 2018;391(10119):462-512.

For peer review only



For peer review only - http://bmjopen.bmj.com/site/about/guidelines.xhtml

7. Hussain M. “Built Environment and Health” What We Create And How It Affects Us? IOSR Journal Of Humanities And Social Science (IOSR-JHSS)2016.

8. Duhl LJ, Sanchez, A.K & World Health Organization. Regional Office for Europe. Healthy cities and the city planning process : a background document on links between health and urban planning. Copenhagen : WHO Regional Office for Europe. 1999.

9. Kent J, Thompson S. Health and the built environment: exploring foundations for a new interdisciplinary profession. J Environ Public Health. 2012;2012:958175.

10. Gurjar BR, Ravindra K, Nagpure AS. Air pollution trends over Indian megacities and their local-to-global implications. Atmospheric Environment. 2016;142:475-95.

11. Giles-Corti B, Vernez-Moudon A, Reis R, Turrell G, Dannenberg AL, Badland H, et al. City planning and population health: a global challenge. Lancet. 2016;388(10062):2912-24.

12. Prüss-Üstün A, Wolf J, Corvalán CF, Bos R, Neira MP. Preventing disease through healthy environments: a global assessment of the burden of disease from environmental risks. Geneva: World Health Organization; 2016 2016.

13. Moher D, Liberati A, Tetzlaff J, Altman DG. Preferred reporting items for systematic reviews and meta-analyses: the PRISMA statement. PLoS Med. 2009;6(7):e1000097.

14. Stang A. Critical evaluation of the Newcastle-Ottawa scale for the assessment of the quality of nonrandomized studies in meta-analyses. Eur J Epidemiol. 2010;25(9):603-5.

15. Herzog R, Álvarez-Pasquin MJ, Díaz C, Del Barrio JL, Estrada JM, Gil Á. Are healthcare workers’ intentions to vaccinate related to their knowledge, beliefs and attitudes? a systematic review. BMC Public Health. 2013;13(1):154.

16. Malambo P KA, De Villiers A, Lambert EV, Puoane T Built Environment, Selected Risk Factors and Major Cardiovascular Disease Outcomes: A Systematic Review. PLoS ONE. 2016;PLoS ONE 11(11): e0166846.

17. den Braver NR, Lakerveld J, Rutters F, Schoonmade LJ, Brug J, Beulens JWJ. Built environmental characteristics and diabetes: a systematic review and meta-analysis. BMC Med. 2018;16(1):12.

18. Cerin E, Nathan A, van Cauwenberg J, Barnett DW, Barnett A, on behalf of the Council on E, et al. The neighbourhood physical environment and active travel in older adults: a systematic review and meta-analysis. International Journal of Behavioral Nutrition and Physical Activity. 2017;14(1):15.

19. Chandrabose M, Rachele JN, Gunn L, Kavanagh A, Owen N, Turrell G, et al. Built environment and cardio-metabolic health: systematic review and meta-analysis of longitudinal studies. Obes Rev. 2019;20(1):41-54.

20. Whitlock MC. Combining probability from independent tests: the weighted Z-method is superior to Fisher's approach. J Evol Biol. 2005;18(5):1368-73.

21. Zhang T. The effects of economic development and built environment on diabetes in CHINA. Popul Health Metr. 2017;15(1):35.

22. Curto A, Ranzani O, Milà C, Sanchez M, Marshall JD, Kulkarni B, et al. Lack of association between particulate air pollution and blood glucose levels and diabetic status in peri-urban India. Environ Int. 2019;131:105033.

23. Wang M, Jin Y, Dai T, Yu C, Zheng S, Nie Y, et al. Association between ambient particulate matter (PM(10)) and incidence of diabetes in northwest of China: A prospective cohort study. Ecotoxicol Environ Saf. 2020;202:110880.

24. Yang Y, Guo Y, Qian ZM, Ruan Z, Zheng Y, Woodward A, et al. Ambient fine particulate pollution associated with diabetes mellitus among the elderly aged 50 years and older in China. Environ Pollut. 2018;243(Pt B):815-23.

25. Lin B-C, Yen Y-T, Lao XQ, Chen Y-H, Chan T-C. Association between neighborhood greenspace and fasting plasma glucose from a large cohort study in Taiwan. Urban Forestry & Urban Greening. 2019;44:126439.



For peer review only - http://bmjopen.bmj.com/site/about/guidelines.xhtml

26. Yang BY, Markevych I, Heinrich J, Bowatte G, Bloom MS, Guo Y, et al. Associations of greenness with diabetes mellitus and glucose-homeostasis markers: The 33 Communities Chinese Health Study. Int J Hyg Environ Health. 2019;222(2):283-90.

27. Leng H, Li S, Yan S, An X. Exploring the Relationship between Green Space in a Neighbourhood and Cardiovascular Health in the Winter City of China: A Study Using a Health Survey for Harbin. Int J Environ Res Public Health. 2020;17(2).

28. Fujiwara T, Takamoto I, Amemiya A, Hanazato M, Suzuki N, Nagamine Y, et al. Is a hilly neighborhood environment associated with diabetes mellitus among older people? Results from the JAGES 2010 study. Soc Sci Med. 2017;182:45-51.

29. Lee H, Kang HM, Ko YJ, Kim HS, Kim YJ, Bae WK, et al. Influence of urban neighbourhood environment on physical activity and obesity-related diseases. Public Health. 2015;129(9):1204-10.

30. Li Y, Mallinson PAC, Bhan N, Turner C, Bhogadi S, Sharma C, et al. Neighborhood physical food environment and cardiovascular risk factors in India: Cross-sectional evidence from APCAPS. Environ Int. 2019;132:105108.

31. Biswas RK, Kabir E. Influence of distance between residence and health facilities on non-communicable diseases: An assessment over hypertension and diabetes in Bangladesh. PLoS One. 2017;12(5):e0177027.

32. Jacob AM, Datta M, Kumpatla S, Selvaraj P, Viswanthan V. Prevalence of Diabetes Mellitus and Exposure to Suspended Particulate Matter. J Health Pollut. 2019;9(22):190608.

33. Chuang KJ, Yan YH, Chiu SY, Cheng TJ. Long-term air pollution exposure and risk factors for cardiovascular diseases among the elderly in Taiwan. Occup Environ Med. 2011;68(1):64-8.

34. Liu C, Yang C, Zhao Y, Ma Z, Bi J, Liu Y, et al. Associations between long-term exposure to ambient particulate air pollution and type 2 diabetes prevalence, blood glucose and glycosylated hemoglobin levels in China. Environ Int. 2016;92-93:416-21.

35. Liang F, Yang X, Liu F, Li J, Xiao Q, Chen J, et al. Long-term exposure to ambient fine particulate matter and incidence of diabetes in China: A cohort study. Environ Int. 2019;126:568-75.

36. Liu T, Chen X, Xu Y, Wu W, Tang W, Chen Z, et al. Gut microbiota partially mediates the effects of fine particulate matter on type 2 diabetes: Evidence from a population-based epidemiological study. Environ Int. 2019;130:104882.

37. Mehdinia SM, Beygi B, Farkhani EM, Taghipour A, Farahmand SK, Moeinian K, editors. Association Between the Prevalence of Diabetes and PM2.5: A Cross-Sectional, Ecological Study2019.

38. Qiu H, Schooling CM, Sun S, Tsang H, Yang Y, Lee RS, et al. Long-term exposure to fine particulate matter air pollution and type 2 diabetes mellitus in elderly: A cohort study in Hong Kong. Environ Int. 2018;113:350-6.

39. Han Y, Wang Y, Li W, Chen X, Xue T, Chen W, et al. Susceptibility of prediabetes to the health effect of air pollution: a community-based panel study with a nested case-control design. Environmental Health. 2019;18(1):65.

40. Khafaie MA, Salvi SS, Yajnik CS, Ojha A, Khafaie B, Gore SD. Air pollution and respiratory health among diabetic and non-diabetic subjects in Pune, India-results from the Wellcome Trust Genetic Study. Environ Sci Pollut Res Int. 2017;24(18):15538-46.

41. Sohn D, Oh H. Gender-dependent Differences in the Relationship between Diabetes Mellitus and Ambient Air Pollution among Adults in South Korean Cities. Iran J Public Health. 2017;46(3):293-300.

42. Chen L, Zhou Y, Li S, Williams G, Kan H, Marks GB, et al. Air pollution and fasting blood glucose: A longitudinal study in China. Sci Total Environ. 2016;541:750-5.

43. Shan A, Zhang Y, Zhang LW, Chen X, Li X, Wu H, et al. Associations between the incidence and mortality rates of type 2 diabetes mellitus and long-term exposure to ambient air pollution: A 12-year cohort study in northern China. Environ Res. 2020;186:109551.



For peer review only - http://bmjopen.bmj.com/site/about/guidelines.xhtml

44. Tong L, Li K, Zhou Q. The association between air pollutants and morbidity for diabetes and liver diseases modified by sexes, ages, and seasons in Tianjin, China. Environ Sci Pollut Res Int. 2015;22(2):1215-9.

45. Wong SF, Yap PS, Mak JW, Chan WLE, Khor GL, Ambu S, et al. Association between long-term exposure to ambient air pollution and prevalence of diabetes mellitus among Malaysian adults. Environ Health. 2020;19(1):37.

46. Liu F, Guo Y, Liu Y, Chen G, Wang Y, Xue X, et al. Associations of long-term exposure to PM1, PM2.5, NO2 with type 2 diabetes mellitus prevalence and fasting blood glucose levels in Chinese rural populations. Environment International. 2019;133:105213.

47. Maas J, Verheij RA, de Vries S, Spreeuwenberg P, Schellevis FG, Groenewegen PP. Morbidity is related to a green living environment. Journal of Epidemiology and Community Health. 2009;63(12):967.

48. Bodicoat DH, Donovan G, Dalton AM, Gray LJ, Yates T, Edwardson C, et al. The association between neighbourhood greenspace and type 2 diabetes in a large cross-sectional study. BMJ Open. 2014;4(12):e006076.

49. Ihlebæk C, Aamodt G, Aradi R, Claussen B, Thorén KH. Association between urban green space and self-reported lifestyle-related disorders in Oslo, Norway. Scandinavian Journal of Public Health. 2018;46(6):589-96.

50. Brown SC, Lombard J, Wang K, Byrne MM, Toro M, Plater-Zyberk E, et al. Neighborhood Greenness and Chronic Health Conditions in Medicare Beneficiaries. American Journal of Preventive Medicine. 2016;51(1):78-89.

51. Clark C, Sbihi H, Tamburic L, Brauer M, Frank LD, Davies HW. Association of Long-Term Exposure to Transportation Noise and Traffic-Related Air Pollution with the Incidence of Diabetes: A Prospective Cohort Study. Environmental Health Perspectives. 2017;125(8):087025.

52. Lee JJ, Hwang SJ, Mutalik K, Corey D, Joyce R, Block JP, et al. Association of built environment characteristics with adiposity and glycaemic measures. Obes Sci Pract. 2017;3(3):333-41.

53. De la Fuente F, Saldías MA, Cubillos C, Mery G, Carvajal D, Bowen M, et al. Green Space Exposure Association with Type 2 Diabetes Mellitus, Physical Activity, and Obesity: A Systematic Review. Int J Environ Res Public Health. 2020;18(1).

54. Urban green space interventions and health: A review of impacts and effectiveness. WHO global site. 2017.

55. Petrişor A-I. IMPERFECT HEALTH: THE MEDICALIZATION OFARCHITECTURE. 2012.

56. Pereira G, Christian H, Foster S, Boruff BJ, Bull F, Knuiman M, et al. The association between neighborhood greenness and weight status: an observational study in Perth Western Australia. Environmental Health. 2013;12(1):49.

57. Valson JS, Kutty VR, Soman B, Jissa VT. Spatial Clusters of Diabetes and Physical Inactivity: Do Neighborhood Characteristics in High and Low Clusters Differ? Asia Pac J Public Health. 2019;31(7):612-21.

58. Paquet C, Coffee NT, Haren MT, Howard NJ, Adams RJ, Taylor AW, et al. Food environment, walkability, and public open spaces are associated with incident development of cardio-metabolic risk factors in a biomedical cohort. Health & Place. 2014;28:173-6.

59. Pradeepa RMAaR. Built environment, physical activity and diabetes. RECENT TRENDS IN DIABETES RESEARCH. 2017;CURRENT SCIENCE, VOL. 113, NO. 7.

60. Bodicoat DH, Carter P, Comber A, Edwardson C, Gray LJ, Hill S, et al. Is the number of fast-food outlets in the neighbourhood related to screen-detected type 2 diabetes mellitus and associated risk factors? Public Health Nutr. 2015;18(9):1698-705.

61. Ahern M, Brown C, Dukas S. A national study of the association between food environments and county-level health outcomes. J Rural Health. 2011;27(4):367-79.

62. Myers CA, Slack T, Broyles ST, Heymsfield SB, Church TS, Martin CK. Diabetes prevalence is associated with different community factors in the diabetes belt versus the rest of the United States. Obesity (Silver Spring). 2017;25(2):452-9.

For peer review only



For peer review only - http://bmjopen.bmj.com/site/about/guidelines.xhtml

63. AlHasan DM, Eberth JM. An ecological analysis of food outlet density and prevalence of type II diabetes in South Carolina counties. BMC Public Health. 2016;16(1):10.
64. Carroll SJ, Paquet C, Howard NJ, Coffee NT, Adams RJ, Taylor AW, et al. Local descriptive body weight and dietary norms, food availability, and 10-year change in glycosylated haemoglobin in an Australian population-based biomedical cohort. BMC Public Health. 2017;17(1):149.
65. Morland K, Diez Roux AV, Wing S. Supermarkets, other food stores, and obesity: the atherosclerosis risk in communities study. Am J Prev Med. 2006;30(4):333-9.
66. Turner C, Kalamatianou S, Drewnowski A, Kulkarni B, Kinra S, Kadiyala S. Food Environment Research in Low- and Middle-Income Countries: A Systematic Scoping Review. Adv Nutr. 2020;11(2):387-97.
67. Yang BY, Fan S, Thiering E, Seissler J, Nowak D, Dong GH, et al. Ambient air pollution and diabetes: A systematic review and meta-analysis. Environ Res. 2020;180:108817.
68. Liu F, Chen G, Huo W, Wang C, Liu S, Li N, et al. Associations between long-term exposure to ambient air pollution and risk of type 2 diabetes mellitus: A systematic review and meta-analysis. Environ Pollut. 2019;252(Pt B):1235-45.
69. Fitzmaurice GM, Ravichandran C. A primer in longitudinal data analysis. Circulation. 2008;118(19):2005-10.
70. Stephens C. The urban environment, poverty and health in developing countries. Health Policy Plan. 1995;10(2):109-21.
71. Tortajada AKBaC. Urbanization and Migration in Developing Asia. The Diplomat. 2015 **September 11, 2015**.
72. Stephania T. Miller DGS, James W. Pichert, and Nasar U. Ahmed. Shaping Environments for Reductions in Type 2 Diabetes Risk Behaviors: A Look at CVD and Cancer Interventions. Diabetes Spectrum. 2002(Diabetes Spectrum. 2002;15(3):176.).



For peer review only - http://bmjopen.bmj.com/site/about/guidelines.xhtml

**Figure1. Study identification, screening, and eligibility, guided by PRISMA**

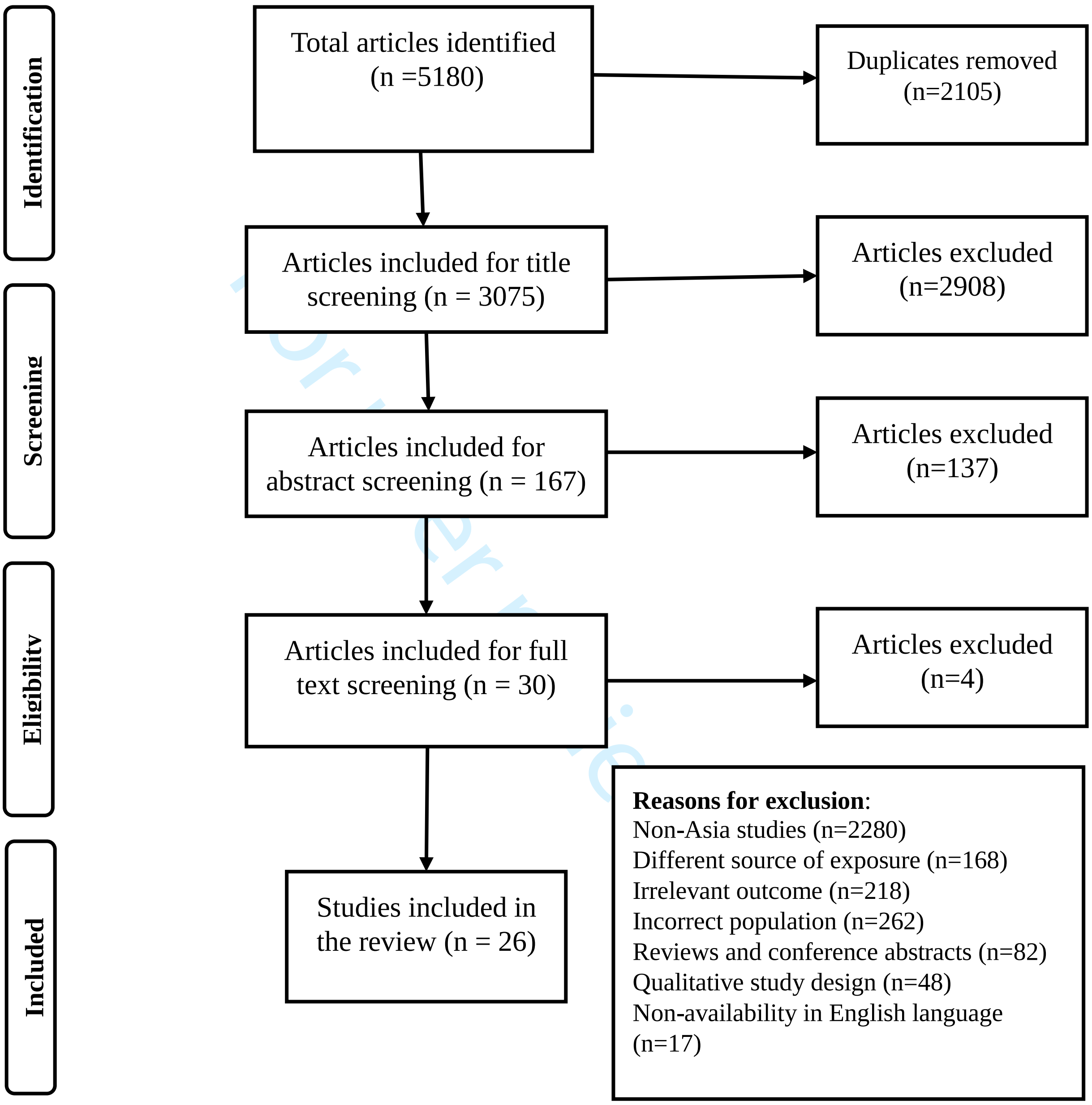

**Online Supplement-1**

**Systematic literature search strategy in Medline, Embase and Global Health using OVID platform**

| Concepts | No. | Term |
|---|---|---|
| Built Environment | 1 | (environment$ adj1 (design$ or green$ or pollut$ or built or food or natur$ or determinant$ or influence or support$ or approach$ or variable$ or attribute$ or barrier$ or local$ or rural$ or urban$ or object$ or perceiv$ or measure$ or obes$ or residen$ characteris$ or correlate$ or soci$ or physical$)).mp. |
| | 2 | (travel$ adj1 (active$ or passive$)).tw. |
| | 3 | (plan$ adj1 (urban$ or town$ or city$)).tw. |
| | 4 | (park$ adj1 (access$ or urban$)).tw. |
| | 5 | (space$ adj1 (green$ or open$ or natur$)).tw. |
| | 6 | (fast food$ adj1 (dens$ or restaurant$)).tw. |
| | 7 | (food$ adj1 (outlet$ or suppl$)).tw. |
| | 8 | (facilit$ adj1 (recreation$ or sport$)).tw. |
| | 9 | (pollut$ adj1 (air$ or noise$)).tw. |
| | 10 | (residen$ adj1 (loca$ or proximit$)).tw. |
| | 11 | retail densit*.tw. |
| | 12 | walkability.mp. |
| | 13 | contextual research.tw. |
| | 14 | sprawl$.ab. |
| | 15 | zoning.ab. |
| | 16 | worksite*.ab. |
| | 17 | cyclability.ab. |
| | 18 | sidewalk$.ab. |
| | 19 | crowd$.ab. |
| | 20 | pedestrian$.ab. |
| | 21 | cyclepath$.ab. |
| | 22 | aesthetic*.ab. |
| | 23 | remote$.ab. |

For peer review only

| | | |
|---|---|---|
| | 24 | neighbo?rhood*.mp. |
| | 25 | 2 or 3 or 4 or 5 or 6 or 7 or 8 or 9 or 10 or 11 or 12 or 13 or 14 or 15 or 16 or 17 or 18 or 19 or 20 or 21 or 22 or 23 or 24 |
| Type 2 T2D mellitus | 26 | hyperglyc$.mp. |
| | 27 | T2D.ab. |
| | 28 | glyc?emi$.mp. |
| | 29 | (glucose adj1 (blood or high or low or $tolerance)).tw. |
| | 30 | insulin resistan$.tw. |
| | 31 | hba1$.mp. |
| | 32 | hyp?glyc$.mp. |
| | 33 | 26 or 27 or 28 or 29 or 30 or 31 or 32 |
| Observational studies | 34 | epidem$.mp. |
| | 35 | disease association.tw. |
| | 36 | inciden$.mp. |
| | 37 | prevalen$.mp. |
| | 38 | 34 or 35 or 36 or 37 |
| Asia | 39 | asia$.mp. |
| | 40 | (Afghanistan$ or Armenia$ or Azerbaijan or Bahrain or Bangladesh$ or Bhutan$ or Brunei$ or Cambodia$ or Chin$ or Cyprus or Georgia$ or India$ or Indonesia$ or Iran$ or Iraq$ or Israel$ or Japan$ or Jordan or Kazakhstan or Kuwait or Kyrgyzstan or Laos or Leban$ or Malaysia$ or Maldives or Mongolia$ or Myanmar or Burm$ or Nepal$ or North Korea$ or Oman or Pakistan$ or Palestin$ or Philippines or Qatar$ or Russia$ or Saudi Arabia$ or Singapor$ or South Korea$ or Sri Lanka$ or Syria$ or Taiwan or Tajikistan or Thailand or Timor-Leste or Turk$ or Turkmenistan or United Arab Emirates or Uzbekistan or Vietnam$ or Yemen).mp. |
| | 41 | 39 or 40 |
| Complete Search | 42 | 1 and 25 and 33 and 38 and 41 |

For peer review only

**Online Supplement-2**

**Key to Quality Assessment of the selected studies**

| Criteria | Items | Description | Maximum Score |
|---|---|---|---|
| **Selection** | **Representativeness of the sample** | The studies choose the samples which were truly or somewhat representative of the average in the target population or not. | 1 |
| | **Study size** | The sample size the study selected was justified and satisfactory or not | 2 |
| | **Ascertainment of the exposure** | If the study applied validated measurement tool to ascertain the risk factors, we assigned two stars. Additionally, we still assigned one star to the study applied non-validated measurement tool which was available or described. | 2 |
| **Comparability** | **The Subjects in Different Outcome Groups are Comparable** | The study controlled for the confounding factors or not | 1 |
| **Outcome measures and analysis** | **Assessment of the outcome** | The study applied independent blind assessment, record linkage, self-report or no description. If it used independent blind assessment or record linkage, we assigned two stars. If it used self-report, we assigned one star. | 2 |
| | **Statistical test** | If the statistical test used to analyze the data was clearly described and appropriate, and the measurement of the association was presented, including confidence intervals and the probability level (i.e., p value), we assigned one star. | 1 |

Based on standard reference Newcastle-Ottawa Quality Assessment scale(15)



For peer review only - http://bmjopen.bmj.com/site/about/guidelines.xhtml

**Quality score for eligible studies**

| S.No | Source | Selection | | | Comparability | Outcome measures and analysis | | Total (9) | Quality |
|---|---|---|---|---|---|---|---|---|---|
| | | Representativeness of the sample | Study size | Ascertainment of the exposure | The subjects in different outcome groups are comparable | Assessment of the outcome | Statistical test | | |
| 1. | Chuang et al 2011(34) | 1 | 2 | 2 | 1 | 2 | 0 | 8 | Very good |
| 2. | Tong et al 2015 (45) | 1 | 2 | 2 | 1 | 2 | 1 | 9 | Very good |
| 3. | Lee et al 2015 (30) | 1 | 2 | 2 | 1 | 2 | 0 | 8 | Very good |
| 4. | Chen et al 2016 (43) | 1 | 2 | 2 | 1 | 2 | 1 | 9 | Very good |
| 5. | Liu Fei et al 2016 (35) | 1 | 2 | 2 | 1 | 2 | 0 | 8 | Very good |
| 6. | Zhang et al 2017 (22) | 1 | 2 | 1 | 1 | 2 | 0 | 7 | Good |
| 7. | Fujiwara et al 2017(29) | 1 | 2 | 2 | 1 | 2 | 0 | 8 | Very good |
| 8. | Biswas and Kabir 2017 (32) | 1 | 2 | 2 | 1 | 2 | 1 | 9 | Very good |
| 9. | Khafaie et al 2017 (41) | 1 | 2 | 2 | 1 | 2 | 0 | 8 | Very good |
| 10. | Sohn and Oh 2017(42) | 1 | 2 | 2 | 1 | 2 | 1 | 9 | Very good |
| 11. | Qiu et al 2018 (39) | 1 | 2 | 2 | 1 | 2 | 1 | 9 | Very good |
| 12. | Liu et al 2019 (47) | 1 | 2 | 2 | 1 | 2 | 1 | 9 | Very good |



For peer review only - http://bmjopen.bmj.com/site/about/guidelines.xhtml

| | | | | | | | | | |
|---|---|---|---|---|---|---|---|---|---|
| 13. | Liang et al 2019 (36) | 1 | 2 | 2 | 1 | 2 | 0 | 8 | Very good |
| 14. | Curto et al 2019 (23) | 1 | 2 | 2 | 1 | 2 | 0 | 8 | Very good |
| 15. | Han et al 2019 (40) | 1 | 1 | 2 | 1 | 2 | 1 | 8 | Very good |
| 16. | Lin et al 2019(26) | 1 | 2 | 2 | 1 | 2 | 1 | 9 | Very good |
| 17. | Yang et al 2019 (25) | 1 | 2 | 2 | 1 | 1 | 1 | 8 | Very good |
| 18. | Jacob et al 2019 (33) | 0 | 1 | 2 | 0 | 2 | 0 | 5 | Satisfactory |
| 19. | Li et al 2019 (31) | 1 | 1 | 2 | 1 | 2 | 1 | 9 | Very good |
| 20. | Tao et al 2019 (37) | 1 | 2 | 2 | 1 | 2 | 1 | 9 | Very good |
| 21. | Yang et al 2019(27) | 1 | 2 | 2 | 1 | 2 | 1 | 9 | Very good |
| 22. | Leng et al 2020 (28) | 1 | 2 | 1 | 1 | 2 | 1 | 8 | Very good |
| 23. | Wong et al 2020 (46) | 1 | 2 | 2 | 1 | 2 | 1 | 9 | Very good |
| 24. | Wang et al 2020 (24) | 1 | 2 | 2 | 1 | 2 | 1 | 9 | Very good |
| 25. | Mehdinia et al 2020 (38) | 1 | 2 | 2 | 1 | 2 | 1 | 9 | Very good |
| 26. | Shan et al 2020 (44) | 1 | 2 | 1 | 1 | 2 | 1 | 8 | Good |

For peer review only

**Online Supplement -3**

**Summary of study characteristics of articles**

| Author, year & country | Study design | Sample size & Age (in years) | Outcome type | Exposure category | Exposure assessment | Study Findings |
|---|---|---|---|---|---|---|
| Chuang et al, 2011, Taiwan (34) | Cross-sectional | 1023 participants aged ≥ 54 years | T2D risk | Air pollution | Secondary | There was a stronger association between T2D and PM2.5 than with two gases, O3 and NO2. |
| Tong et al, 2015, China(45) | Cross-sectional | 1430 participants from all age group | T2D risk | Air pollution | Secondary | Increase in 2-day average concentrations of particulate matters like (PM10), SO2, and NO2 correspond to increases in T2D morbidity. The association was in expected direction |
| Lee et al, 2015, South Korea(30) | Cross-sectional | 16178 participants aged ≥20 years | T2D prevalence | Walkability | Secondary | T2D mellitus is associated with walkable environments, and physical activity may contribute to the association. |
| Chen et al, 2016, China(43) | Cohort | 27685 participants aged between18-98 years | T2D prevalence | Air pollution | Secondary | In women, elderly and overweight people, air pollutants had a higher effect on FBG than in males, young and underweight people. This suggests that air pollution may increase the levels of FBG. |
| Liu et al, 2016, China(35) | Cross-sectional | 11847 participants aged ≥45 years | T2D prevalence and risk | Air pollution | GIS | Significant increases in T2D prevalence, fasting glucose levels, and hba1c levels were associated with long-term exposure to PM2.5. |

For peer review only

| | | | | | | |
|---|---|---|---|---|---|---|
| Zhang et al, 2017, China (22) | Cross-sectional | 2745 participants aged ≥18 years | T2D Prevalence | Accessibility to services | Secondary | China's T2D prevalence is inextricably linked to the built environment as well as individual economic factors. When combined with other influential factors, China's strong economic growth has made obesity and T2D more common. |
| Fujiwara et al, 2017, Japan (29) | Cross-sectional | 8904 participants aged ≥ 65 years | T2D Prevalence | Food environment and walkability | GIS | There was no association between hilly neighbourhoods and T2D mellitus, but they protected against poorly managed T2D mellitus. |
| Biswas and Kabir, 2017, Bangladesh (32) | Cross-sectional | 7544 participants aged ≥35 years | T2D Prevalence | Availability and accessibility to services | Secondary | It was found that T2D was not significantly associated with distance from a health facility. |
| Khafaie et al, 2017, India(41) | Cross-sectional | 1213 participants aged ≥46 years | T2D risk | Air pollution | Atmospheric dispersion model | Exposure to PM10 long-term correlates with high insulin resistance and high glycaemia. |
| Sohn and Oh, 2017, South Korea(42) | Cross-sectional | 43941 participants with median age 46.7 | T2D prevalence | Air pollution | Secondary | In South Korea, PM10 and SO2 levels may be associated with T2D prevalence, but there appears to be a gender difference. Exposures to either PM10 or SO2 were significantly related to the prevalence of DM 2 among women, but not among men. |
| Qiu et al, 2018, China(39) | Cohort | 53905 participants aged ≥65 years | T2D incidence and | Air pollution | GIS | In Hong Kong's elderly population, long-term exposure to high levels of PM2.5 may increase the prevalence and incidence of type 2 T2D mellitus. |

| | | | | | | |
|---|---|---|---|---|---|---|
| | | | prevalence | | | |
| Liu et al, 2019, China(47) | Cohort | 39191 participants aged between 18-79 years | T2D prevalence and risk | Air pollution | GIS | There is a positive correlation between higher exposures of air pollutants and increased odds of developing type 2 T2D, along with higher fasting blood sugar levels among Chinese rural residents. |
| Liang et al, 2019, China(36) | Cohort | 88397 participants with mean age 51.7 | T2D incidence | Air pollution | GIS | There is evidence that prolonged exposure to PM2.5 is associated with T2D incidence in China. If air quality is improved in China, it will impact the reduction of T2D incidence in China. |
| Curto et al, 2019, India (23) | Cross-sectional | 5065 participants aged ≥18 years | T2D Prevalence | Air pollution | Gravimetric sampler | Women's blood glucose levels were negatively influenced by PM2.5 and black carbon (BC). In men, associations were negative for PM2.5 and positive for BC. |
| Han et al, 2019, China(40) | Case-control | 120 participants in each group aged between 50-65 years | T2D prevalence | Air pollution | Particulate sampler | People with T2D are more susceptible to the acute cardio metabolic effects of air pollution than healthy people. |
| Lin et al, 2019, Taiwan (26) | Cross-sectional | 773602 participants aged ≥ 18 years | T2D risk | Urban green space | GIS | Residential greenness appears to be associated with lower glucose levels. Physical activity is a possible mediator of this association. |
| Yang et al, 2018, China(25) | Cross-sectional | 15477 participants aged ≥ 50 years | T2D prevalence | Air pollution | Secondary | T2D and PM2.5 are statistically significantly associated. In stratified analyses by fruit consumption, a lower association was found among participants. |

| | | | | | | |
|---|---|---|---|---|---|---|
| Jacob et al, 2019, India (33) | Cross-sectional | 410 participants aged ≥18 years | T2D prevalence | Air pollution | Gravimetric sampler | People living in areas with high particulate matter exposure were 77.5% more likely to have T2D than those living in areas with less exposure. |
| Li et al, 2019, India(31) | Cross-sectional | 5764 participants with median age 28.8 | T2D risk | Food environment | GIS | Having fewer fruit and vegetable vendors and having more take-out and highly processed foods was associated with higher cardiovascular risk profiles. |
| Tao et al, 2019, China(37) | Cross-sectional | 6627 participants aged ≥18 years | T2D risk | Air pollution | GIS | PM2.5 and PM1 were both linked to an increased risk of impaired fasting glucose (IFG) and T2D. |
| Yang et al., 2019, China(27) | Cross-sectional | 15,477 participants aged 18-74 years | T2D Prevalence | Urban green space | GIS | Higher residential greenness appears to be beneficially associated with both T2D prevalence and glucose-homeostasis markers in Chinese adults. |
| Leng et al, 2020, China(28) | Cross-sectional | 4155 participants aged ≥20 years | T2D prevalence | Urban green space | Secondary | Green space characteristics of a neighborhood were not associated with T2D. |
| Wong et al, 2020, Malaysia (46) | Cross-sectional | 56710 participants from all age group | T2D risk | Air pollution | Secondary | In Malaysia, long-term exposure to O3 is a significant factor associated with under diagnosed DM. The risk of DM may be influenced by the presence of PM10, NO2 and NOx, and their roles should be explored in further detail with other interaction models. |
| Wang et al, 2020, | Cohort | 19884 participants aged between | T2D incidence | Air pollution | Secondary | T2D incidence increased by 17% per 10μg/m3 increase in environmental PM10, and the risk rises gradually with higher PM10 concentrations. The |

For peer review only

| | | | | | | |
|---|---|---|---|---|---|---|
| China(24) | | 20-79 | | | | risk of T2D incidence associated with ambient PM10 was higher among females, young to middle-aged people, overweight and obese subjects, and those with a FPG level at baseline less than 5.6 mmol/L. |
| Mehdinia et al, 2020, Iran(38) | Cross-sectional | 482833 participants aged between 30-59 years | T2D prevalence | Air pollution | Secondary | T2D prevalence and PM2.5 concentrations show a significant association. |
| Shan et al, 2020, China(44) | Cohort | 38529 participants with mean age 44.1 | T2D incidence | Air pollution | Secondary | Among Chinese adults, it was found that long-term exposure to high levels of PM10, SO2, and NO2 increased risk of T2DM incidence and mortality. |

For peer review only

# PRISMA2020Checklist

| Section and Topic | Item # | Checklist item | Location where item is reported |
|---|---|---|---|
| **TITLE** | | | |
| Title | 1 | Identify the report as a systematic review. | Page 1 |
| **ABSTRACT** | | | |
| Abstract | 2 | See the PRISMA 2020for Abstracts checklist. | Page 2 |
| **INTRODUCTION** | | | |
| Rationale | 3 | Describe the rationale for the review in the context of existing knowledge. | Page 4 |
| Objectives | 4 | Provide an explicit statement of the objective(s) or question(s) the review addresses. | Page 5 |
| **METHODS** | | | |
| Eligibility criteria | 5 | Specify the inclusion and exclusion criteria for the review and how studies were grouped for the syntheses. | Page 5, Table 1 |
| Information sources | 6 | Specify all databases, registers, websites, organizations, reference lists and other sources searched or consulted to identify studies. Specify the date when each source was last searched or consulted. | Page 7 |
| Search strategy | 7 | Present the full search strategies for all databases, registers and websites, including any filters and limits used. | Online supplement File 1 |
| Selection process | 8 | Specify the methods used to decide whether a study met the inclusion criteria of the review, including how many reviewers screened each record and each report retrieved, whether they worked independently, and if applicable, details of automation tools used in the process. | Page 6 |
| Data collection process | 9 | Specify the methods used to collect data from reports, including how many reviewers collected data from each report, whether they worked independently, any processes for obtaining or confirming data from study investigators, and if applicable, details of automation tools used in the process. | Page 7-8 |
| Data items | 10a | List and define all outcomes for which data were sought. Specify whether all results that were compatible with each outcome domain in each study were sought(e.g. for all measures, time points, analyses),and if not, the methods used to decide which results to collect. | Online supplement File 3 |
| | 10b | List and define all other variables for which data were sought(e.g. participant and intervention characteristics ,funding sources).Describe any assumptions made about any missing or unclear information. | Page 7-8 |
| Study risk of bias assessment | 11 | Specify the methods used to assess risk of bias in the included studies, including details of the tool(s) used, how many reviewers assessed each study and whether they worked independently, and if applicable, details of automation tools used in the process. | Page 7 |
| Effect measures | 12 | Specify for each outcome the effect measure(s)(e.g. risk ratio, mean difference) used in the synthesis or presentation of results. | Online supplement File 3 |
| Synthesis methods | 13a | Describe the processes used to decide which studies were eligible for each synthesis(e.g. tabulating the study intervention characteristics and comparing against the planned groups for each synthesis(item#5)). | Page 8-9, Online supplement file 2 |
| | 13b | Describe any methods required to prepare the data for presentation or synthesis, such as handling of missing summary statistics, or data conversions. | NA |

For peer review only

PRISMA2020Checklist

| | | | |
|---|---|---|---|
| | 13c | Describe any methods used to tabulate or visually display results of individual studies and syntheses. | Online supplement file 3 |
| | 13d | Describe any methods used to synthesize results and provide a rationale for the choice(s). If meta-analysis was performed, describe the model(s), method(s) to identify the presence and extent of statistical heterogeneity, and software package(s) used. | Page 8-9 |
| | 13e | Describe any methods used to explore possible causes of heterogeneity among study results (e.g. subgroup analysis, meta-regression). | NA |
| | 13f | Describe any sensitivity analyses conducted to assess robustness of the synthesized results. | NA |
| Reporting bias assessment | 14 | Describe any methods used to assess risk of bias due to missing results in a synthesis (arising from reporting biases). | NA |
| Certainty assessment | 15 | Describe any methods used to assess certainty (or confidence) in the body of evidence for an outcome. | NA |

For peer review only

# PRISMA2020Checklist

| Sectionand Topic | Item # | Checklist item | Location whereitem isreported |
|---|---|---|---|
| **RESULTS** | | | |
| Studyselection | 16a | Describetheresults ofthesearchandselection process,from thenumberofrecordsidentifiedin thesearch tothenumber ofstudiesincludedin thereview,ideallyusingaflowdiagram. | Page 11-12, Figure 1 |
| | 16b | Cite studiesthatmightappearto meettheinclusioncriteria,butwhichwereexcluded, andexplainwhy theywereexcluded. | NA |
| Study characteristics | 17 | Cite eachincludedstudyandpresent itscharacteristics. | Online supplement file 3 |
| Riskofbias in studies | 18 | Present assessmentsofriskofbias foreach includedstudy. | NA |
| Resultsof individualstudies | 19 | For all outcomes, present, foreachstudy:(a)summary statisticsforeach group(whereappropriate) and(b)aneffectestimateanditsprecision (e.g.confidence/credibleinterval),ideallyusingstructured tablesor plots. | Online supplement file 3 |
| Resultsof syntheses | 20a | For eachsynthesis, brieflysummarisethecharacteristicsandrisk ofbiasamongcontributing studies. | NA |
| | 20b | Presentresults ofallstatisticalsynthesesconducted.Ifmeta-analysiswasdone,presentforeach thesummaryestimateand its precision(e.g. confidence/credible interval) andmeasuresofstatistical heterogeneity.Ifcomparinggroups, describe the directionoftheeffect. | Page 16-17 |
| | 20c | Presentresults ofallinvestigationsofpossiblecausesofheterogeneityamong studyresults. | NA |
| | 20d | Presentresults ofallsensitivity analysesconducted toassess therobustness ofthesynthesizedresults. | NA |
| Reportingbiases | 21 | Present assessmentsofriskofbias due tomissingresults(arising fromreportingbiases)foreachsynthesisassessed. | NA |
| Certainty of evidence | 22 | Present assessmentsofcertainty(orconfidence) in thebodyofevidence foreach outcomeassessed. | NA |
| **DISCUSSION** | | | |
| Discussion | 23a | Provideageneralinterpretation oftheresults in thecontextof otherevidence. | Page 17-19 |
| | 23b | Discussanylimitationsoftheevidenceincluded inthereview. | Page 20 |
| | 23c | Discussanylimitationsofthereviewprocesses used. | Page 20 |
| | 23d | Discussimplications oftheresults for practice, policy,andfutureresearch. | Page 20-21 |
| **OTHER INFORMATION** | | | |
| Registrationand protocol | 24a | Provideregistrationinformation for thereview,includingregister name andregistration number,or statethatthereviewwas notregistered. | Page 6 |
| | 24b | Indicatewherethereview protocolcan beaccessed, orstatethataprotocolwas notprepared. | Page 6 |
| | 24c | Describeandexplain anyamendments toinformationprovided atregistrationor in theprotocol. | NA |
| Support | 25 | Describesources offinancialor non-financialsupportforthereview,andtheroleofthefunders orsponsorsin thereview. | Page 21 |
| Competing interests | 26 | Declare anycompetinginterestsofreview authors. | NA |
| Availability of data,code and othermaterials | 27 | Reportwhichofthe followingare publiclyavailableandwhere theycanbe found:templatedatacollectionforms;dataextracted from included studies; datausedfor allanalyses;analyticcode;anyothermaterialsusedinthereview. | NA |

For peer review only

For peer review only - http://bmjopen.bmj.com/site/about/guidelines.xhtml

*From:* PageMJ,McKenzieJE,BossuytPM,BoutronI,HoffmannTC,MulrowCD,etal.ThePRISMA2020statement:anupdatedguidelineforreportingsystematicreviews.BMJ2021;372:n71.doi:

10.1136/bmj.n71

# PRISMA2020Checklist

For moreinformation,visit: http://www.prisma-statement.org/

For peer review only

BMJ Open

# Associations of the built environment with Type 2 diabetes in Asia: A systematic review

| | |
|---|---|
| Journal: | *BMJ Open* |
| Manuscript ID | bmjopen-2022-065431.R1 |
| Article Type: | Original research |
| Date Submitted by the Author: | 28-Feb-2023 |
| Complete List of Authors: | Raveendiran, Aarthi; Madras Diabetes Research Foundation, Research Operations; SRM Institute of Science and Technology<br>Mehreen, T S; Imperial College London, School of Public Health; Madras Diabetes Research Foundation, Research Operations<br>Moosawi, Suzana; Imperial College London Faculty of Medicine, Epidemiology<br>Kusuma, Dian; Imperial College Business School, Centre for Health Economics and Policy Innovations<br>Harish, Ranjani; Madras Diabetes Research Foundation, Translational Research<br>Pradeepa, Rajendra; Madras Diabetes Research Foundation<br>Venkatasubramanian, Padma; SRM Institute of Science and Technology<br>Mohan, V; Madras Diabetes Research Foundation<br>Anjana, Ranjit Mohan; Madras Diabetes Research Foundation; SRM Institute of Science and Technology<br>Fecht , D ; Imperial College London, MRC Centre for Environment and Health, School of Public Health |
| <b>Primary Subject Heading</b>: | Epidemiology |
| Secondary Subject Heading: | Public health, Diabetes and endocrinology |
| Keywords: | EPIDEMIOLOGY, Health & safety < HEALTH SERVICES ADMINISTRATION & MANAGEMENT, PREVENTIVE MEDICINE, PUBLIC HEALTH, General diabetes < DIABETES & ENDOCRINOLOGY |
| | |

SCHOLARONE™
Manuscripts

I, the Submitting Author has the right to grant and does grant on behalf of all authors of the Work (as defined in the below author licence), an exclusive licence and/or a non-exclusive licence for contributions from authors who are: i) UK Crown employees; ii) where BMJ has agreed a CC-BY licence shall apply, and/or iii) in accordance with the terms applicable for US Federal Government officers or employees acting as part of their official duties; on a worldwide, perpetual, irrevocable, royalty-free basis to BMJ Publishing Group Ltd ("BMJ") its licensees and where the relevant Journal is co-owned by BMJ to the co-owners of the Journal, to publish the Work in this journal and any other BMJ products and to exploit all rights, as set out in our licence.

The Submitting Author accepts and understands that any supply made under these terms is made by BMJ to the Submitting Author unless you are acting as an employee on behalf of your employer or a postgraduate student of an affiliated institution which is paying any applicable article publishing charge ("APC") for Open Access articles. Where the Submitting Author wishes to make the Work available on an Open Access basis (and intends to pay the relevant APC), the terms of reuse of such Open Access shall be governed by a Creative Commons licence – details of these licences and which Creative Commons licence will apply to this Work are set out in our licence referred to above.

Other than as permitted in any relevant BMJ Author's Self Archiving Policies, I confirm this Work has not been accepted for publication elsewhere, is not being considered for publication elsewhere and does not duplicate material already published. I confirm all authors consent to publication of this Work and authorise the granting of this licence.

For peer review only

# Associations of the built environment with Type 2 diabetes in Asia: A systematic review

Garudam Raveendiran Aarthi [1,2,3], Thaharullah Shah Mehreen [1,3], Suzana Al Moosawi[3], Dian Kusuma[4], Harish Ranjani[5], Rajendra Pradeepa [1], Venkatasubramanian Padma[2], Viswanathan Mohan[6], Ranjit Mohan Anjana[2,6], Daniela Fecht[7,8]

1. Department of Research Operations, Madras Diabetes Research Foundation, Chennai, India

2. School of Public Health, SRM Institute of Science and Technology, Kattankulathur, India

3. School of Public Health, Imperial College London, London, UK

4. Centre for Health Economics & Policy Innovation, Imperial College Business School, London, UK

5. Department of Translational Research, Madras Diabetes Research Foundation, Chennai, India

6. Department of Diabetology, Madras Diabetes Research Foundation, Chennai, India

7. MRC Centre for Environment and Health, Department of Epidemiology and Biostatistics, School of Public Health, Imperial College London, London, UK

8. National Institute for Health Research Health Protection Research Unit on Chemical and Radiation Threats and Hazards, Department of Epidemiology and Biostatistics, School of Public Health, Imperial College London, London, UK

## ADDRESS FOR CORRESPONDENCE

**Dr Daniela Fecht**
MRC Centre for Environment and Health,
School of Public Health,
Imperial College London,
Norfolk Place
London W2 1PG, UK
Email: d.fecht@imperial.ac.uk

**Dr Ranjit Mohan Anjana**
Madras Diabetes Research Foundation,
4, Conran Smith Road, Gopalapuram,
Chennai- 600086, INDIA
Phone: 91-44-4396 8888
Fax: 91-44-28350935

For peer review only

Email: dranjana@drmohans.com

For peer review only

## ABSTRACT

**Objectives:** Our study aimed to systematically review the literature and synthesise findings on potential associations of built environment characteristics with type 2 diabetes (T2D) in Asia.

**Design:** Systematic review of the literature.

**Data Sources:** Online databases Medline, Embase, and Global Health were used to identify peer-reviewed journal articles published from inception to 23rd January 2023.

**Eligibility criteria:** Eligible studies included cohort, cross sectional, and case control studies that explored associations of built environment characteristics with T2D among adults 18 years and older in Asia.

**Data extraction and synthesis:** Covidence Online was used to remove duplicates and perform title, abstract and full text screening. Data extraction was carried out by two independent reviewers using the OVID database and data was imported into MS Excel. Out of 5,208 identified studies, 28 studies were included in this systematic review. Due to heterogeneity in study design, built environment and outcome definitions, a semi-qualitative analysis was conducted, which synthesised results using weighted *z*-scores.

**Results:** Five broad categories of built environment characteristics were associated with T2D in Asia. These included urban green space, walkability, food environment, availability and accessibility of services such as recreational and healthcare facilities and air pollution. We found very strong evidence of a positive association of particulate matter ($PM_{2.5}$, $PM_{10}$), nitrogen dioxide and sulfur dioxide ($p$<0.001) with T2D risk.

**Conclusion:** Several built environment attributes were significantly related to T2D in Asia. When compared to Western countries, very few studies have been conducted in Asia. Further research is, therefore, warranted to establish the importance of the built environment on T2D.



For peer review only - http://bmjopen.bmj.com/site/about/guidelines.xhtml

For peer review only

Such evidence is essential for public health and planning policies to (re)design neighborhoods and help improve public health across Asian countries.

**Registration:** This systematic review was conducted according to PRISMA guidelines and registered with PROSPERO database (CRD42020214852).

**Keywords:** diabetes, epidemiology, health and safety, preventive medicine, public health

For peer review only



For peer review only - http://bmjopen.bmj.com/site/about/guidelines.xhtml

**Strengths and Limitations**

- This is the first systematic review to synthesise the evidence base on association of built environmental characteristics including urban green space, walkability, food environments, availability and accessibility to services (recreational and healthcare), and air pollution with Type 2 diabetes in Asia.
- Due to the heterogeneity in built environment characteristics, outcome measures and methodology used in the eligible studies, a formal meta-analysis was not feasible.
- The weighted *z*-test was used to quantify the strength of evidence and synthesise the findings, but only selected air pollutants were assessed due to lack of studies on other categories.

For peer review only



For peer review only - http://bmjopen.bmj.com/site/about/guidelines.xhtml

## INTRODUCTION

Non-communicable diseases (NCDs) are a key cause of poor health globally, and account for seven out of ten deaths worldwide. In low- and middle-income countries (LMICs), NCDs are a major barrier to development (1). Type 2 diabetes (T2D) is a large contributor to the NCD burden and a major risk factor for cardiovascular disease, affecting the quality of life and reducing life expectancy by about 4 to 8 years compared to those without the disease (2). T2D is characterised by insufficient insulin production and inefficient insulin utilisation by the body (3) and reduction in β cell function (4). Approximately 537 million people worldwide are affected by T2D and the number is predicted to rise to 783 million by 2045 (5, 6). LMICs are expected to see an 87% increase in T2D prevalence, with the Indian subcontinent predicted to experience the largest increase (7). More than 60% of people with T2D in the world are Asians (8).

T2D is primarily explained by genetic, demographic, and lifestyle factors. Recent findings, however, indicate that an increasing proportion of T2D is attributable to environmental factors including the built environment (1,9). The built environment encompasses the physical and built context in which people live, work and socialize, including man-made structures such as buildings and transportation systems, and natural features such as parks and urban forests (10,11). The built environment can have both positive and negative impacts on T2D. Factors such as ambient air pollution or a transport network which discourages active transport can have negative impacts on health (12,13). Key built environment characteristics which improve public health include environments which promote physical activity, healthy eating and low pollution. The growth of cities without planned infrastructure to facilitate healthy lifestyle choices constrains opportunities for improved public health (14). With healthier environments, 23% of deaths could be prevented worldwide (15).

For peer review only



For peer review only - http://bmjopen.bmj.com/site/about/guidelines.xhtml

Approximately 4.7 billion people live in Asia, representing 60% of the world's population (16). Yet, most of the empirical evidence on the built environment and T2D comes from Western countries which have a very different urban fabric compared to Asian cities. Asian cities are characterised by rapid, often unstructured, urban growth, high population density and, in some cases, temporal mix of land use(17). To better understand the relationship between the built environment and T2D in Asia, it is, therefore, important to synthesise the available evidence in the Asian context, to identify gaps in knowledge and support local city planning and public health interventions.

## METHODS AND ANALYSIS

### Search strategy

A comprehensive systematic bibliographic search was conducted to identify epidemiological evidence on the associations of built environment characteristics with T2D in Asian countries. The systematic review followed the Preferred Reporting Items for Systematic Reviews and Meta-Analysis (PRISMA) guidelines (18). The protocol was registered with the International Prospective Register of Systematic Reviews (PROSPERO) database (CRD42020214852). A comprehensive search strategy identified peer-reviewed journal articles from inception to 23rd January 2023 using the electronic bibliographic databases Medline, Embase and Global Health. The search strategy included combinations of keywords related to the built environment (urban green space, walkability, food environment, accessibility of services, air pollution, density and clustering measures), health outcome (T2D), study design (observational studies including cohort, cross-sectional and case-control studies) and study area (Asia). A reference search was carried out against newly identified articles until no more relevant articles were discovered.



For peer review only - http://bmjopen.bmj.com/site/about/guidelines.xhtml

**Patient and Public Involvement:** There was no direct involvement of patients or the public in this study.

**Eligibility criteria**

Study titles and abstracts were examined to assess their eligibility for inclusion based on predefined participant, intervention, comparison, and outcome (PICO) criteria, shown in Table1. Only studies published in the English language were considered. As T2D accounts for the majority of diabetes cases in the general community (>90 %) (3), studies that did not specify the type of diabetes were included.

For peer review only

**Table 1. Inclusion and exclusion criteria for literature search**

| | **Inclusion criteria** | **Exclusion criteria** |
|---|---|---|
| *Population* | Adults aged 18 years and above residing in Asia. Studies conducted on adults including specific population such as pregnant women were included. | Animal studies and those involving specific populations such as pregnant women. |
| *Intervention* | Studies exploring any of the following built environment characteristics:<br>• Urban green space, including parks, ground cover vegetation, street trees, green roofs; | Those that do not meet the criteria of built environment features and studies in an experimental setting. |



For peer review only - http://bmjopen.bmj.com/site/about/guidelines.xhtml

| | | |
|---|---|---|
| | • Walkability, including land-use mix, residential density, street connectivity;<br>• Food environment, including distance and density of health and unhealthy foot outlets;<br>• Availability and accessibility to services, including distance and density of shops, health care and recreational facilities;<br>• Environmental pollutants: noise, air pollution. | |
| *Comparators* | NA | NA |
| *Outcome* | Studies reporting outcomes on: prevalence/incidence of T2D and pre-diabetes, fasting blood glucose (FBG) and 2-hour plasma glucose (2hPG), glycated haemoglobin (HbA1c), insulin and Homeostatic model assessment of insulin resistance (HOMA-IR) levels. | Studies that evaluated admissions to hospital or emergency departments due to T2D. |
| *Study type* | Observational studies on the association of built environment features with T2D outcomes: including cohort, cross sectional and case-control studies. | Controlled trials, reviews, case reports and interventional studies. Specific publications which do not report original |


For peer review only - http://bmjopen.bmj.com/site/about/guidelines.xhtml

| | | |
|---|---|---|
| | | scientific research including letters, editorials, interviews, and legal documents. |

137

138 Access and availability of urban green space and higher walkability which encourages people
139 to walk in their local community, are both hypothesised to increase physical activity and
140 consequently reduce T2D prevalence. The food environment facilitates either healthy or
141 unhealthy food acquisition and consumption within the wider food system and can, therefore,
142 have both positive and negative effects on T2D. Accessibility and availability of services
143 including recreational facilities and healthcare services are hypothesized to decrease T2D
144 prevalence. Long-term air pollution linked to local sources including traffic has been
145 hypothesised to increase T2D prevalence.

146 **Screening, data extraction and preparation**

147 Online Supplement File 1 provides a comprehensive overview of the search terms, including
148 MESH terms used for MEDLINE, Embase, and Global Health using OVID databases and
149 extracted data using Microsoft Excel. Duplicates were removed and studies screened against
150 the pre-defined study selection criteria, independently by two reviewers (GRA and TSM),
151 using Covidence, an online, systematic review tool. At all screening stages, title, abstract and
152 full text, discrepancies were resolved through discussion with a mediator (HR). A standardised
153 data extraction form was used for collection of information on methodology, outcome and
154 exposure measures, including study characteristics (year of publication, study design, sample
155 size, country), participant characteristics (gender, age range), health outcomes (T2D outcome
156 and measurement method), built environment characteristics (type, measurement tool), and



For peer review only - http://bmjopen.bmj.com/site/about/guidelines.xhtml

statistical methods. Data extraction was performed by reviewer TSM and data extraction was assessed by reviewer GRA.

**Quality assessment**

To assess the quality of included studies, the Newcastle - Ottawa Quality Assessment Scale (NOS) for Observational Cohort in an adapted version for cross-sectional studies (19) was used, following previous examples from the built environment literature (20–22) . The quality assessment was performed by reviewers GRA and TSM, conflicts were discussed with reviewer HR and resolved. Each study was scored for quality by blinding the authors, institutions, country, and journal. In evaluating the quality of each study, six criteria were used: (i) representativeness of the sample, (ii) study size, (iii) ascertainment of the exposure, (iv) comparable subjects in different outcome groups, (v) assessment of the outcome, (vi) statistical test. Based on these criteria, star ratings were awarded. The six criteria were categorized into three broader categories relating to: a) the study population, b) the comparability of population and c) characterization of exposure/outcome variables (23). The maximum number of stars awarded for the study population was 5 stars while the comparability of populations and characterization of exposure/outcome variables were awarded 1 star and 3 stars, respectively. The study quality was evaluated based on the total number of stars assigned to each study, with eight to nine stars defined as “very good” quality, six to seven stars defined as “good” quality, four to five stars defined as “satisfactory” quality, and zero to four stars defined as “unsatisfactory” quality. Online Supplement File 2 provides the summary of quality assessment using the NOS.

**Data synthesis**

Due to the heterogeneity in built environment characteristics, outcome measures and methodology used in the eligible studies, a formal meta-analysis was not feasible.



For peer review only - http://bmjopen.bmj.com/site/about/guidelines.xhtml

Heterogeneity in built environment studies is a common issue that has been described by other authors (20,22,24). To overcome this issue, a semi-quantitative meta-analytical method was used which has been developed and described in detail previously (25,26). This method synthesizes studies based on a weighted *z*-test, which considers the direction of findings, either in the expected or unexpected direction, and weights their level of significance based on the *p*-value (27) as shown in Table 2.

**Table 2. Significance levels and associated z-values used in semi-quantitative meta-analysis**

| Statistically significant | *α* | *z*-value |
|---|---|---|
| Yes (expected direction) | 0.05 | 1.96 |
| | 0.1 | 1.64 |
| No (null) | 0 | 0 |
| Yes (unexpected direction) | 0.1 | - 1.64 |
| | 0.05 | - 1.96 |

In order to calculate the *z*-score associated with a specific exposure–outcome relationship, the sum of *z*-values was divided by the square root of the sum of squared weights, using the quality assessment scores of individual studies as weights. After calculating the weighted *z* -value, the two-tailed *p*-value was used to determine whether the evidence was strong or weak using the following criteria: $p<0.05$ for weak evidence, $p<0.01$ for strong evidence, and $p<0.001$ for very strong evidence. The following equation was used to derive a summarized weighted *z* -value:

For peer review only



For peer review only - http://bmjopen.bmj.com/site/about/guidelines.xhtml

$$Weightedz = \frac{\sum weight_j z_j}{\sqrt{\sum weight_j^2}}$$

## RESULTS

Initially, 5,208 articles were identified, of which 2,116 were duplicates. After screening of articles according to eligibility criteria, 28 articles were eligible for inclusion in the systematic review (Figure 1).

The majority of studies were conducted in China (53.6%), followed by India (17.8%), South Korea (7.1%), Japan (3.6%), Taiwan (3.6%), Bangladesh (3.6%) and South Asia [Bangladesh and Sri Lanka] (3.6%). The earliest study was published in 2011, over 75% of studies after 2017. Cross-sectional study designs were used in 71% of the studies, while cohort study designs and case control were used in 25% and 4% of the studies, respectively. All studies included both male and female participants. Sample sizes ranged from 120 to 341,211 participants, and different methods for participant recruitment and data collection were used such as medical records and self-reported health status. Study sample sizes <1,000 were used by 7%, and studies using sample sizes 1,001-2,500 and >2,500 were used by 11% and 82%, respectively. The quality score ranged from 5 to 9. The mean quality score was 8.3 (standard deviation 0.85) indicating overall good and very good quality. The detailed characteristics from all the studies included in the systematic review are presented in Online Supplement File 3.

The majority of studies used objectively collected data on diabetes ($n$ = 21), with the outcome being evaluated by biomedical tests or retrieved from registry records. One study used self-reported data (28) and three studies used a combination of hospital records and self-reported data (29-31). The outcomes included diabetes and pre-diabetes prevalence and incidence ($n$ = 19), levels of fasting blood glucose (FBG) ($n$ = 11), 2-hour plasma glucose (2hPG) ($n$ = 1), glycated hemoglobin (HbA1c) ($n$ = 5), insulin level ($n$ = 1), and homeostatic model assessment



For peer review only - http://bmjopen.bmj.com/site/about/guidelines.xhtml

of insulin resistance (HOMA-IR) ($n$ = 1). Outcome variables were expressed as changes in continuous measurements in longitudinal or cohort studies (for example, changes in FBG levels from baseline to follow-up) or as T2D prevalence in cross sectional studies.

Studies explored a wide range of built environment characteristics on i) urban green space ($n$ = 3), ii) walkability ($n$ = 2), iii) food environment ($n$ = 3), iv) availability and accessibility to services ($n$ = 2), and v) air pollution ($n$ =19). Studies used either objectively measured exposure derived using geographic information systems (GIS) ($n$ = 15) or audit measures ($n$ = 13). The sphere of influence of exposures was defined as either a circular buffer ($n$ = 3) or street-network buffer ($n$ = 1) around the participants' place of residence, ranging from 400 meters to 1,600 meters. Some studies also used proximity to destinations ($n$ = 2) and modeled air pollution concentration at place of residences ($n$ = 8).

## Built environment and T2D

The results are presented for each built environment category separately. Study characteristics and quality assessment for all included studies are presented in Online Supplement File 2 and 3, respectively. Due to fewer than 5 studies on most built environment categories, the weighted $z$- score was calculated only for air pollution.

### Urban green space

Three studies investigated the association of urban green space with T2D (32-34), two studies conducted in China (33.34) and one in Taiwan (32). All studies used FBG as a measure of T2D; one study used 2-h glucose, HOMA-IR (33). Greenness was assessed with Normalized Difference Vegetative Index (NDVI) (32,33), or soil adjusted vegetation index (SAVI) (33), one



For peer review only - http://bmjopen.bmj.com/site/about/guidelines.xhtml

study assessed green space ratio, green vision index, and evergreen tree configuration (34). The statistical analysis was performed using logistic regression (32), binary logistic regression (33) and linear mixed effect models (32,34). Greenness was associated with lower glucose levels in Taiwan (32) and China (33) ($p<0.05$). Physical activity (32), body mass index (BMI) and air pollution (33) were considered as potential mediators for this association. However, the other exposure variables used to measure urban green space did not show an association with T2D (34).

**Walkability**

Two studies assessed walkability using a cross-sectional design in Japan (35) and South Korea (36). Both studies assessed T2D using FBG levels, one study assessed HbA1c levels, and medical history, with study participants recruited from secondary data (35). The study conducted in Japan assessed walkability using the availability of parks, slope, and land use mix, while the study from South Korea used street patterns, slopes, and neighborhood amenities, such as the amount of neighborhood park space, number of shopping malls, and distance between communities and malls, which were both quantified and qualitatively assessed through site observations and GIS analyses. Multilevel regression (35) and multiple logistic regression (36) were used to perform the statistical analysis. According to the study conducted in South Korea, people who lived in communities with better street networks, defined as grid layout of pedestrian pathways, and pleasant landscape, including street trees, had a lower risk of T2D (odds ratio (OR): 0.83, 95% confidence intervals (CI): 0.77 to 0.91). Other associations of exposure variables with T2D were not statistically significant. The study from Japan, did not find a statistically significant association of a hilly environment on T2D. Steeper slopes, on the other hand, reduced the risk of poorly controlled T2D (HbA1c levels of ≥7.5% in healthy people without any co-morbidity or ≥8% in the elderly).



For peer review only - http://bmjopen.bmj.com/site/about/guidelines.xhtml

## Food environment

The food environment was examined by three studies (35,37,38). Among participants aged 65 and older, one study examined the perception of access to grocery stores for fresh fruits and vegetables in Japan (35). The South Asian Biobank study which analysed data from Bangladesh and Sri Lanka, collected information on fast food restaurants, super markets, mobile carts and stationary carts within 300 meter buffer around participants' place of residence (38). The study conducted in India, collected information on shops and services selling food, tobacco, and alcohol, as well as the density of vendors. All studies assessed T2D based on FBG levels, while two studies additionally assessed T2D with HbA1c and insulin levels (35.37) and the South Asian Biobank study additionally used high glucose levels and diagnosed diabetes. The study from Japan, found no significant association between perception of access to grocery stores and T2D. Using three levels of mixed-effect linear regression, the density and distance of fruit and vegetable vendors and highly processed/takeout food vendors were analysed in relation to T2D in the Indian study. The South Asian Biobank study used ordinary least squares multivariate regression analysis to examined in association with T2D among adults and its heterogeneity according to income and gender. The associations between highly processed and take-away food vendor density and FBG (OR: 0.30, 95% CI: -0.14 to 0.74) and insulin (OR: 0.13, 95% CI: -0.04 to 0.29) were statistically non-significant (37). There was no association between distance to the nearest fruit and vegetable vendor or highly processed and take-away food vendor and FBG or insulin levels (35). An increase in blood glucose levels and the likelihood of being diagnosed with diabetes was associated with having at least one Fast food restaurants (FFR) locally by 16% (OR: 1.6, 95% CI: 1.01 to 1.33) and 19% (OR: 1.19, 95% CI: 1.03 to 1.38), respectively. FFR density had a stronger positive association with blood glucose levels among women than men. In contrast, FFR proximity had



For peer review only - http://bmjopen.bmj.com/site/about/guidelines.xhtml

a stronger association with blood glucose levels among men and those with higher incomes(38).

**Availability and accessibility to services**

Two studies conducted in China (28) and Bangladesh (39) examined the relationship between the availability and accessibility of services such as healthcare, recreational facilities and T2D. Both studies used a cross sectional study design and collected data from medical records. T2D was measured using FBG and self-reported T2D. Generalized linear mixed models (39) and ordinary least squares models (28) were used for statistical analyses. T2D was more prevalent in areas of greater distance from health facilities, such as community clinics, but the difference was statistically not significant (OR: 1.05, 95% CI: 0.6 to 1.61) (28). In the study conducted in Bangladesh, the association of distance to services with T2D was statistically non-significant except for the distance to a cinema hall (OR: 1.76, 95% CI: 1.04 to 2.98).

**Air pollution**

Nineteen studies assessed air pollution in relation to T2D. Particulate matter (PM) of various particle sizes, black carbon (BC), nitrogen dioxide ($NO_2$), ozone ($O_3$), and sulphur dioxide ($SO_2$) were the main air pollutants studied. Eleven studies were cross-sectional, followed by cohort studies (*n* = 7) and one case-control studies. There were 12 studies conducted in China, three in India, and one each in South Korea, Iran, Taiwan, and Malaysia. 482,833 people were enrolled in a cross-sectional study with the largest sample size (32). Particulate matter $PM_{2.5}$ (*n* = 12) (29,31,40–48) was the most commonly assessed air pollutant in relation to T2D, followed by $PM_{10}$ (*n* = 8) (30,41,49–54), $NO_2$ (*n* = 6) (41,51–55), $SO_2$ (*n* = 6) (49–54), $NO_x$ (*n*



For peer review only - http://bmjopen.bmj.com/site/about/guidelines.xhtml

= 2) (49,54), $O_3$ ($n$ = 2) (41,54), $PM_1$ ($n$ = 2) (44,55) and BC ($n$ = 1) (29). Six studies used medical history and T2D treatments to assess T2D status, while other studies used FBG, HbA1c, and fasting plasma glucose levels, and one study used random capillary blood glucose to assess T2D. Several studies used secondary data such as data from Korea Community Health Survey data, Malaysian National Health, China Health and Retirement Longitudinal survey and Morbidity Surveys for health. Air pollution exposure was assessed using either measurements from local environmental monitoring centers and gravity samples, or air pollution models such as satellite-based spatial statistical models, spatio-temporal models, and AERMOD dispersion models.

Six studies reported that there was a significant positive association between air pollution and T2D among females but not in males (30,43,46,50,51,53). Two studies performed gender-specific stratified subgroup analyses and found a positive association for females but not for males (46,50). In stratified analyses, exposure to air pollution has been associated with an increase in diabetes incidence among under 65 years compared to the elderly (30,43,53), rural subjects in comparison to urban subjects (43), BMI <25 in comparison to BMI ≥25 (53), non-smokers in comparison to smokers (43), and normotensive in comparison to hypertensive (30,43). There is also evidence that air pollution has positive associations with T2D among the elderly (51), overweight and obese individuals (30).

Twelve studies evaluated $PM_{2.5}$ with eleven reporting a positive association between air pollution and T2D and one study reported a negative association (39). Most studies indicate that the risk of T2D increased when exposure to $PM_{10}$ ($n$=8) or $SO_2$ ($n$=6) increased. One study reported no association between $PM_{10}$ and T2D in men (55). Six studies on $NO_2$ found a significant increase in T2D risk when exposed to higher levels $NO_2$.

Using the $z$- test, we synthesized the evidence for $PM_{2.5}$, $PM_{10}$, $NO_2$, and $SO_2$, separately. The two-tailed $p$-value from the weighted $z$-value for $PM_{2.5}$, $PM_{10}$, $NO_2$ and $SO_2$ and T2D was



For peer review only - http://bmjopen.bmj.com/site/about/guidelines.xhtml

p<0.001, indicating a very strong association. The number of studies for $NO_x$, ozone, $PM_1$ and BC was too small to derive *z* statistics.

## DISCUSSION

This is the first systematic review to synthesise the evidence of the association of built environmental characteristics including urban green space, walkability, food environments, availability and accessibility to services (recreational and healthcare), and air pollution with T2D in Asia. We found strong evidence of an association of air pollutants such as $PM_{2.5}$, $PM_{10}$, $NO_2$, and $SO_2$ with higher incidence/risk/prevalence of T2D. Other built environment categories lacked consistent evidence to establish an association with T2D.

We found that measures for urban green space including green space ratio, green vision index, and evergreen configuration were not significantly associated with T2D (28,34,39). In contrast to our findings, some cross-sectional studies in Western countries found lower rates of T2D among people exposed to more green spaces, including studies from the Netherlands (56) and the United Kingdom (57). A Norwegian study, however, did not find an association between green space and T2D, which the authors argued might be due to the fact that most of the residents had easy access to green spaces (58). Similarly to results of two studies conducted in Taiwan (32) and China (33), A systematic review conducted globally to examine the association between greenness as measured by NDVI and T2D supported the notion that people and communities exposed to greenness, especially in their neighborhood, have a lower risk of T2D (59). South Asia's urban green space has been steadily diminishing. Green spaces and conservation areas are currently under threat, primarily because of unplanned and disorganised housing construction in cities (60).



For peer review only - http://bmjopen.bmj.com/site/about/guidelines.xhtml

Walkability is hypothesized to increase physical activity and therefore might contribute to lower T2D rates. An effective neighborhood design considers how land-use decisions affect people's access to essential services in their communities. Completing, connecting and compacting a neighbourhood has been associated with health benefits such as increased physical activity, safer and easier transportation, enhanced employment productivity, and social inclusion. Strategies to encourage connectivity include enhancing walkability, establishing mixed land use, and designing complete and compact neighborhoods (61). An Australian study indicated that hilly neighborhoods may prevent T2D (62),while the study conducted in Japan in a hilly neighborhood, showed no link between walkability and T2D (35). This difference might be due to the specific population group which focused on the elderly in the Japan study. Higher built-up density and reduced greenness were found to be connected with lower T2D rates and physical inactivity in Kerala, India (63). A study in South Korea found that participants in communities that have better street networks like grid pattern of pedestrian sidewalks and attractive scenery were less likely to have T2D (36). This is in line with findings from the Australian study indicating that study participants who lived in areas with better walkability were less likely to develop T2D (64). Our systematic review found that there is currently not much research carried out on particular built environmental attributes related to accessibility to healthcare centers in Asia. The need for longitudinal studies is thus imperative to identify such specific built environment attributes that may influence T2D outcomes in order to establish future urban design regulations for healthier and more efficient communities. The effects of walking in neighborhoods can be increased by providing pedestrian paths that are usable and unobstructed, by implementing motor-traffic reduction strategies, by increasing good street connectivity, and by providing parks, green space, playgrounds and recreation areas (65).



For peer review only - http://bmjopen.bmj.com/site/about/guidelines.xhtml

Our systematic review found consistent evidence of an association of the food environment with T2D risk/prevalence. An increase in fruit and vegetable vendor availability was associated with a lower T2D risk in an Indian study (37). In this study, there was strong evidence for an association between T2D risk and food vendor density within 400 metres of the place of residence, while the association between take-away/processed food vendor availability and T2D was not significant. Two studies conducted in America and the United Kingdom discovered a link between increased number of fast-food restaurants and convenience stores and a higher prevalence of T2D (66,67). Four studies conducted in the United States and Australia found no significant association between supermarket/grocery store availability and T2D prevalence (67–71).Various socio-cultural and economic norms influence how people purchase and consume food, which may explain differences in the association between food availability and T2D risk in different countries (72).

We found consistent and strong association between air pollution and T2D. Higher levels of $PM_{10}$, $PM_{2.5}$ were associated with an increased risk of T2D incidence and prevalence; higher $NO_2$ exposure was associated with a higher T2D prevalence, but no statistically significant association with $O_3$ was found. In a previous systematic review on air pollution and T2D, with most studies from North America and Europe (73), results were consistent with our study. Another meta-analysis showed consistent evidence of high exposure to air pollution being linked with greater T2D risk/prevalence (74) but identified a lack of studies from LMICs.

According to previous studies conducted in Western countries (75–78), there is stronger evidence of associations between air pollution and T2D among women compared to men. Clougherty et al., suggests that sex-linked biological differences in hormonal complement, body size, activity patterns, or co-exposures may explain such differences (79). Women have been found to suffer more severe health effects due to air pollution, particularly among the



For peer review only - http://bmjopen.bmj.com/site/about/guidelines.xhtml

elderly or when assessing residential exposure (79). There is further evidence that women are more susceptible to air pollution due to hyperresponsive airways (80), lung size, airway diameter (81) and residential environmental factors (82). A Danish Nurse cohort study reported that $PM_{2.5}$ and diabetes incidence increase in never-smokers and are more prominent in obese individuals (76).

In this systematic review, we identified a paucity of evidence from a majority of countries in Asia. In particular, studies from Southeast Asia, including Indonesia, the fourth most populous country in the world, are lacking. Only seven cohort studies were conducted using a nationally representative sample while the other studies selected samples from specific regions such as specific cities, towns or counties. Hence, this systematic review might not be representative of the Asian population. Most of the studies in our review measured built environment attributes at a single point in time, however, longitudinal exposure data can be beneficial to detect changes in exposure in relation to changes is T2D (83).

Overall, evidence from LMICs in Asia on the relationship between the built environment and T2D is still lacking. Despite the vast amount of literature exploring the traditional risk factors for NCDs, little attention has been directed towards specific factors of the built environment related to T2D outcomes in Asian populations. Depending on the quality and characteristics of the built environment, there can be large differences between developed and developing countries, which can affect T2D patterns among Asian residents (84). The urbanisation trend in Asia is expected to undergo a significant shift in the next decade. In light of this circumstance, urban planners have a crucial opportunity to design smart cities and environments that encourage an healthy lifestyles among their residents (85). Nevertheless, this prime target cannot be achieved without a profound evidence base of the relationship between



For peer review only - http://bmjopen.bmj.com/site/about/guidelines.xhtml

built environment and health and wellbeing (86). Understanding the role that the built environment plays in influencing disease risk factors may help prevent the onset or deterioration of T2D with consequently vast benefits for public health.

## CONCLUSION

Our systematic review has identified several built environment characteristics which were significantly related to T2D in Asia, in particular air pollution. Results also highlight potential effect modification by age and sex. Future studies should assess the modifying role of socioeconomic status and ethnicity. Such studies should be carefully designed to better understand the role of potential confounders, risk factors and effect modifiers. This particularly applies to LMIC wherein India and China already have a huge burden from T2D. Such evidence is essential for public health and planning policies to (re)design neighborhoods and help improve public health across Asian countries.

**Ethical Approval:** Ethical approval was not necessary because no human or animal samples were used. This review used only secondary data.

**Contributor ship Statement:** A search strategy was developed and guided by GRA, TSM, DK, SAM and DF, while independent searches were conducted by GRA and TSM, and conflicts were resolved by HR. The data was interpreted by SAM, DK, and RP. After data synthesis and analysis, a draft of the manuscript was prepared by GRA, shared with PV, VM, ARM and DF for their input. Corrections were made based on their suggestions. The accuracy and integrity of the work are investigated and resolved by ARM and DF. Corresponding authors DF and ARM corrected and approved the final version for submission.

**Competing Interest:** None declared.

**Funding:** This work was supported by the MRC Centre for Environment and Health, which is currently funded by the Medical Research Council (MR/S019669/1, 2019-2024). This study is



For peer review only - http://bmjopen.bmj.com/site/about/guidelines.xhtml

part funded by the National Institute for Health Research (NIHR) Health Protection Research Unit in Chemical and Radiation Threats and Hazards, a partnership between UK Health Security Agency and Imperial College London. Infrastructure support for the Department of Epidemiology and Biostatistics was provided by the NIHR Imperial Biomedical Research Centre. The first author (GRA) worked with the ICL team as a visiting researcher in the School of Public Health as part of a training programme funded by the National Institute for Health and Care Research (NIHR's) Short Placement Award for Research Collaboration(NIHR GHR SPARC PILOT 01-19-06). The second author (TSM) was a student at ICL who pursued her M.Sc Epidemiology in the School of Public Health, entirely funded by the National Institute for Health and Care Research- Global Health Research Unit (NIHR GHRU) project. This research was also supported by the National Institute for Health Research (NIHR) (16/136/68) using UK aid from the UK Government to support global health research. The views expressed in this publication are those of the authors and not necessarily those of the NIHR,UK Health Security Agency or the UK Department of Health and Social Care.

**Data sharing statement:** All data relevant to the study are included in the article or uploaded as supplementary information.

## References

1. GBD 2015 Risk Factors Collaborators. Global, regional, and national comparative risk assessment of 79 behavioural, environmental and occupational, and metabolic risks or clusters of risks, 1990-2015: a systematic analysis for the Global Burden of Disease Study 2015. Lancet Lond Engl. 2016 Oct 8;388(10053):1659–724.

2. The Emerging Risk Factors Collaboration. Diabetes mellitus, fasting blood glucose concentration, and risk of vascular disease: a collaborative meta-analysis of 102 prospective studies. Lancet. 2010 Jun 26;375(9733):2215–22.

3. Diabetes [Internet]. [cited 2022 Nov 23]. Available from: https://www.who.int/news-room/fact-sheets/detail/diabetes

4. Mahler RJ, Adler ML. Type 2 Diabetes Mellitus: Update on Diagnosis, Pathophysiology, and Treatment. J Clin Endocrinol Metab. 1999 Apr 1;84(4):1165–71.

5. IDF DIABETES ATLAS [Internet]. 10th ed. Brussels: International Diabetes Federation; 2021 [cited 2022 Oct 13]. (IDF Diabetes Atlas). Available from: http://www.ncbi.nlm.nih.gov/books/NBK581934/

6. Saeedi P, Petersohn I, Salpea P, Malanda B, Karuranga S, Unwin N, et al. Global and regional diabetes prevalence estimates for 2019 and projections for 2030 and 2045: Results from the International Diabetes Federation Diabetes Atlas, 9th edition. Diabetes Res Clin Pract [Internet].



For peer review only - http://bmjopen.bmj.com/site/about/guidelines.xhtml

2019 Nov 1 [cited 2022 Feb 18];157. Available from: https://www.diabetesresearchclinicalpractice.com/article/S0168-8227(19)31230-6/fulltext

7. Abate N, Chandalia M. Ethnicity, type 2 diabetes & migrant Asian Indians. Indian J Med Res. 2007 Mar;125(3):251–8.

8. Hu FB. Globalization of Diabetes. Diabetes Care. 2011 Jun;34(6):1249–57.

9. Landrigan PJ, Fuller R, Acosta NJR, Adeyi O, Arnold R, Basu N (Nil), et al. The Lancet Commission on pollution and health. The Lancet. 2018 Feb 3;391(10119):462–512.

10. Hussain M. "Built Environment and Health" What We Create And How It Affects Us? :6.

11. Duhl LJ, Sanchez AK, Europe WHORO for. Healthy cities and the city planning process : a background document on links between health and urban planning [Internet]. Copenhagen: WHO Regional Office for Europe; 1999 [cited 2022 Feb 18]. Report No.: EUR/ICP/CHDV 03 04 03. Available from: https://apps.who.int/iris/handle/10665/108252

12. Kent J, Thompson S. Health and the built environment: exploring foundations for a new interdisciplinary profession. J Environ Public Health. 2012;2012:958175.

13. Gurjar BR, Ravindra K, Nagpure AS. Air pollution trends over Indian megacities and their local-to-global implications. Atmos Environ. 2016 Oct 1;142:475–95.

14. Giles-Corti B, Vernez-Moudon A, Reis R, Turrell G, Dannenberg AL, Badland H, et al. City planning and population health: a global challenge. Lancet Lond Engl. 2016 Dec 10;388(10062):2912–24.

15. Prüss-Üstün A, Wolf J, Corvalán CF, Bos R, Neira MP. Preventing disease through healthy environments: a global assessment of the burden of disease from environmental risks [Internet]. World Health Organization; 2016 [cited 2022 Feb 18]. 147 p. Available from: https://apps.who.int/iris/handle/10665/204585

16. Population of Asia. 2023 demographics: density, ratios, growth rate, clock, rate of men to women. [Internet]. [cited 2023 Jan 21]. Available from: https://www.populationof.net/asia/

17. Chen TL, Chiu HW, Lin YF. How do East and Southeast Asian Cities Differ from Western Cities? A Systematic Review of the Urban Form Characteristics. Sustainability. 2020 Jan;12(6):2423.

18. Moher D, Liberati A, Tetzlaff J, Altman DG, Group TP. Preferred Reporting Items for Systematic Reviews and Meta-Analyses: The PRISMA Statement. PLOS Med. 2009 Jul 21;6(7):e1000097.

19. Wells G, Shea B, O'Connell D, Robertson J, Peterson J, Welch V, et al. The Newcastle-Ottawa Scale (NOS) for Assessing the Quality of Nonrandomized Studies in Meta- Analysis.

20. Herzog R, Álvarez-Pasquin MJ, Díaz C, Del Barrio JL, Estrada JM, Gil Á. Are healthcare workers' intentions to vaccinate related to their knowledge, beliefs and attitudes? a systematic review. BMC Public Health. 2013 Feb 19;13(1):154.

21. Malacarne D, Handakas E, Robinson O, Pineda E, Saez M, Chatzi L, et al. The built environment as determinant of childhood obesity: A systematic literature review. Obes Rev. 2022;23(S1):e13385.



For peer review only - http://bmjopen.bmj.com/site/about/guidelines.xhtml

22. den Braver NR, Lakerveld J, Rutters F, Schoonmade LJ, Brug J, Beulens JWJ. Built environmental characteristics and diabetes: a systematic review and meta-analysis. BMC Med. 2018 Jan 31;16(1):12.

23. Stang A. Critical evaluation of the Newcastle-Ottawa scale for the assessment of the quality of nonrandomized studies in meta-analyses. Eur J Epidemiol. 2010 Sep;25(9):603–5.

24. Malambo P, Kengne AP, Villiers AD, Lambert EV, Puoane T. Built Environment, Selected Risk Factors and Major Cardiovascular Disease Outcomes: A Systematic Review. PLOS ONE. 2016 Nov 23;11(11):e0166846.

25. Cerin E, Nathan A, van Cauwenberg J, Barnett DW, Barnett A, on behalf of the Council on Environment and Physical Activity (CEPA) – Older Adults working group. The neighbourhood physical environment and active travel in older adults: a systematic review and meta-analysis. Int J Behav Nutr Phys Act. 2017 Feb 6;14(1):15.

26. Chandrabose M, Rachele JN, Gunn L, Kavanagh A, Owen N, Turrell G, et al. Built environment and cardio-metabolic health: systematic review and meta-analysis of longitudinal studies. Obes Rev Off J Int Assoc Study Obes. 2019 Jan;20(1):41–54.

27. Whitlock MC. Combining probability from independent tests: the weighted Z-method is superior to Fisher's approach. J Evol Biol. 2005 Sep;18(5):1368–73.

28. Zhang T. The effects of economic development and built environment on diabetes in China. Popul Health Metr. 2017 Sep 29;15(1):35.

29. Curto A, Ranzani O, Milà C, Sanchez M, Marshall JD, Kulkarni B, et al. Lack of association between particulate air pollution and blood glucose levels and diabetic status in peri-urban India. Environ Int. 2019 Oct;131:105033.

30. Wang M, Jin Y, Dai T, Yu C, Zheng S, Nie Y, et al. Association between ambient particulate matter (PM10) and incidence of diabetes in northwest of China: A prospective cohort study. Ecotoxicol Environ Saf. 2020 Oct 1;202:110880.

31. Yang Y, Guo Y, Qian ZM, Ruan Z, Zheng Y, Woodward A, et al. Ambient fine particulate pollution associated with diabetes mellitus among the elderly aged 50 years and older in China. Environ Pollut Barking Essex 1987. 2018 Dec;243(Pt B):815–23.

32. Lin BC, Yen YT, Lao XQ, Chen YH, Chan TC. Association between neighborhood greenspace and fasting plasma glucose from a large cohort study in Taiwan. Urban For Urban Green. 2019 Aug;44:126439.

33. Yang BY, Markevych I, Heinrich J, Bowatte G, Bloom MS, Guo Y, et al. Associations of greenness with diabetes mellitus and glucose-homeostasis markers: The 33 Communities Chinese Health Study. Int J Hyg Environ Health. 2019 Mar;222(2):283–90.

34. Leng H, Li S, Yan S, An X. Exploring the Relationship between Green Space in a Neighbourhood and Cardiovascular Health in the Winter City of China: A Study Using a Health Survey for Harbin. Int J Environ Res Public Health. 2020 Jan;17(2):513.

35. Fujiwara T, Takamoto I, Amemiya A, Hanazato M, Suzuki N, Nagamine Y, et al. Is a hilly neighborhood environment associated with diabetes mellitus among older people? Results from the JAGES 2010 study. Soc Sci Med 1982. 2017 Jun;182:45–51.



For peer review only - http://bmjopen.bmj.com/site/about/guidelines.xhtml

36. Lee H, Kang HM, Ko YJ, Kim HS, Kim YJ, Bae WK, et al. Influence of urban neighbourhood environment on physical activity and obesity-related diseases. Public Health. 2015 Sep;129(9):1204–10.

37. Li Y, Mallinson PAC, Bhan N, Turner C, Bhogadi S, Sharma C, et al. Neighborhood physical food environment and cardiovascular risk factors in India: Cross-sectional evidence from APCAPS. Environ Int. 2019 Nov;132:105108.

38. Kusuma D, Atanasova P, Pineda E, Anjana RM, Silva LD, Hanif AA, et al. Food environment and diabetes mellitus in South Asia: A geospatial analysis of health outcome data. PLOS Med. 2022 Apr 26;19(4):e1003970.

39. Biswas RK, Kabir E. Influence of distance between residence and health facilities on non-communicable diseases: An assessment over hypertension and diabetes in Bangladesh. PLoS ONE. 2017 May 18;12(5):e0177027.

40. Jacob AM, Datta M, Kumpatla S, Selvaraj P, Viswanthan V. Prevalence of Diabetes Mellitus and Exposure to Suspended Particulate Matter. J Health Pollut. 2019 May 22;9(22):190608.

41. Chuang KJ, Yan YH, Chiu SY, Cheng TJ. Long-term air pollution exposure and risk factors for cardiovascular diseases among the elderly in Taiwan. Occup Environ Med. 2011 Jan;68(1):64–8.

42. Liu C, Yang C, Zhao Y, Ma Z, Bi J, Liu Y, et al. Associations between long-term exposure to ambient particulate air pollution and type 2 diabetes prevalence, blood glucose and glycosylated hemoglobin levels in China. Environ Int. 2016;92–93:416–21.

43. Liang F, Yang X, Liu F, Li J, Xiao Q, Chen J, et al. Long-term exposure to ambient fine particulate matter and incidence of diabetes in China: A cohort study. Environ Int. 2019 May;126:568–75.

44. Liu T, Chen X, Xu Y, Wu W, Tang W, Chen Z, et al. Gut microbiota partially mediates the effects of fine particulate matter on type 2 diabetes: Evidence from a population-based epidemiological study. Environ Int. 2019 Sep 1;130:104882.

45. Mehdinia SM, Beygi B, Farkhani EM, Taghipour A, Farahmand SK, Moeinian K. Association Between the Prevalence of Diabetes and PM2.5: A Cross-Sectional, Ecological Study. 2020;7(1).

46. Qiu H, Schooling CM, Sun S, Tsang H, Yang Y, Lee RSY, et al. Long-term exposure to fine particulate matter air pollution and type 2 diabetes mellitus in elderly: A cohort study in Hong Kong. Environ Int. 2018 Apr;113:350–6.

47. Han Y, Wang Y, Li W, Chen X, Xue T, Chen W, et al. Susceptibility of prediabetes to the health effect of air pollution: a community-based panel study with a nested case-control design. Environ Health. 2019 Jul 15;18(1):65.

48. Liu C, Cao G, Li J, Lian S, Zhao K, Zhong Y, et al. Effect of long-term exposure to PM2.5 on the risk of type 2 diabetes and arthritis in type 2 diabetes patients: Evidence from a national cohort in China. Environ Int. 2023 Jan 1;171:107741.

49. Khafaie MA, Salvi SS, Yajnik CS, Ojha A, Khafaie B, Gore SD. Air pollution and respiratory health among diabetic and non-diabetic subjects in Pune, India-results from the Wellcome Trust Genetic Study. Environ Sci Pollut Res Int. 2017 Jun;24(18):15538–46.



For peer review only - http://bmjopen.bmj.com/site/about/guidelines.xhtml

50. Sohn D, Oh H. Gender-dependent Differences in the Relationship between Diabetes Mellitus and Ambient Air Pollution among Adults in South Korean Cities. Iran J Public Health. 2017 Mar;46(3):293–300.

51. Chen L, Zhou Y, Li S, Williams G, Kan H, Marks GB, et al. Air pollution and fasting blood glucose: A longitudinal study in China. Sci Total Environ. 2016 Jan 15;541:750–5.

52. Shan A, Zhang Y, Zhang L wen, Chen X, Li X, Wu H, et al. Associations between the incidence and mortality rates of type 2 diabetes mellitus and long-term exposure to ambient air pollution: A 12-year cohort study in northern China. Environ Res. 2020 Jul;186:109551.

53. Tong L, Li K, Zhou Q. The association between air pollutants and morbidity for diabetes and liver diseases modified by sexes, ages, and seasons in Tianjin, China. Environ Sci Pollut Res Int. 2015 Jan;22(2):1215–9.

54. Wong SF, Yap PS, Mak JW, Chan WLE, Khor GL, Ambu S, et al. Association between long-term exposure to ambient air pollution and prevalence of diabetes mellitus among Malaysian adults. Environ Health Glob Access Sci Source. 2020 Apr 3;19(1):37.

55. Liu F, Guo Y, Liu Y, Chen G, Wang Y, Xue X, et al. Associations of long-term exposure to PM1, PM2.5, NO2 with type 2 diabetes mellitus prevalence and fasting blood glucose levels in Chinese rural populations. Environ Int. 2019 Dec;133(Pt B):105213.

56. Maas J, Verheij RA, de Vries S, Spreeuwenberg P, Schellevis FG, Groenewegen PP. Morbidity is related to a green living environment. J Epidemiol Community Health. 2009 Dec;63(12):967–73.

57. Bodicoat DH, O'Donovan G, Dalton AM, Gray LJ, Yates T, Edwardson C, et al. The association between neighbourhood greenspace and type 2 diabetes in a large cross-sectional study. BMJ Open. 2014 Dec 23;4(12):e006076.

58. Ihlebæk C, Aamodt G, Aradi R, Claussen B, Thorén KH. Association between urban green space and self-reported lifestyle-related disorders in Oslo, Norway. Scand J Public Health. 2018 Aug;46(6):589–96.

59. De la Fuente F, Saldías MA, Cubillos C, Mery G, Carvajal D, Bowen M, et al. Green Space Exposure Association with Type 2 Diabetes Mellitus, Physical Activity, and Obesity: A Systematic Review. Int J Environ Res Public Health. 2021 Jan;18(1):97.

60. Urban green space interventions and health: A review of impacts and effectiveness. Full report [Internet]. 2022 [cited 2022 Nov 29]. Available from: https://www.who.int/europe/publications/m/item/urban-green-space-interventions-and-health--a-review-of-impacts-and-effectiveness.-full-report

61. Petrisor AI. Imperfect Health: The Medicalization of Architecture. IAFOR J Sustain Energy Environ. 2015 Feb 1;2.

62. Pereira G, Christian H, Foster S, Boruff BJ, Bull F, Knuiman M, et al. The association between neighborhood greenness and weight status: an observational study in Perth Western Australia. Environ Health Glob Access Sci Source. 2013 Jun 19;12:49.

63. Valson JS, Kutty VR, Soman B, Jissa VT. Spatial Clusters of Diabetes and Physical Inactivity: Do Neighborhood Characteristics in High and Low Clusters Differ? Asia Pac J Public Health. 2019 Oct;31(7):612–21.



For peer review only - http://bmjopen.bmj.com/site/about/guidelines.xhtml

For peer review only

64. Paquet C, Coffee NT, Haren MT, Howard NJ, Adams RJ, Taylor AW, et al. Food environment, walkability, and public open spaces are associated with incident development of cardio-metabolic risk factors in a biomedical cohort. Health Place. 2014 Jul;28:173–6.

65. Anjana RM, Pradeepa R. Built environment, physical activity and diabetes. Curr Sci. 2017;113(7):1327–36.

66. Bodicoat DH, Carter P, Comber A, Edwardson C, Gray LJ, Hill S, et al. Is the number of fast-food outlets in the neighbourhood related to screen-detected type 2 diabetes mellitus and associated risk factors? Public Health Nutr. 2015 Jun;18(9):1698–705.

67. Ahern M, Brown C, Dukas S. A national study of the association between food environments and county-level health outcomes. J Rural Health Off J Am Rural Health Assoc Natl Rural Health Care Assoc. 2011;27(4):367–79.

68. Myers CA, Slack T, Broyles ST, Heymsfield SB, Church TS, Martin CK. Diabetes prevalence is associated with different community factors in the diabetes belt versus the rest of the United States. Obes Silver Spring Md. 2017 Feb;25(2):452–9.

69. AlHasan DM, Eberth JM. An ecological analysis of food outlet density and prevalence of type II diabetes in South Carolina counties. BMC Public Health. 2016 Jan 5;16:10.

70. Carroll SJ, Paquet C, Howard NJ, Coffee NT, Adams RJ, Taylor AW, et al. Local descriptive body weight and dietary norms, food availability, and 10-year change in glycosylated haemoglobin in an Australian population-based biomedical cohort. BMC Public Health. 2017 Feb 2;17(1):149.

71. Morland K, Diez Roux AV, Wing S. Supermarkets, other food stores, and obesity: the atherosclerosis risk in communities study. Am J Prev Med. 2006 Apr;30(4):333–9.

72 Turner C, Kalamatianou S, Drewnowski A, Kulkarni B, Kinra S, Kadiyala S. Food Environment Research in Low- and Middle-Income Countries: A Systematic Scoping Review. Adv Nutr Bethesda Md. 2020 Mar 1;11(2):387–97.

73. Yang BY, Fan S, Thiering E, Seissler J, Nowak D, Dong GH, et al. Ambient air pollution and diabetes: A systematic review and meta-analysis. Environ Res. 2020 Jan;180:108817.

74. Liu F, Chen G, Huo W, Wang C, Liu S, Li N, et al. Associations between long-term exposure to ambient air pollution and risk of type 2 diabetes mellitus: A systematic review and meta-analysis. Environ Pollut Barking Essex 1987. 2019 Sep;252(Pt B):1235–45.

785. Eze IC, Hemkens LG, Bucher HC, Hoffmann B, Schindler C, K ünzli N, et al. Association between Ambient Air Pollution and Diabetes Mellitus in Europe and North America: Systematic Review and Meta-Analysis. Environ Health Perspect. 2015 May;123(5):381–9.

76. Hansen AB, Ravnskjær L, Loft S, Andersen KK, Bräuner EV, Baastrup R, et al. Long-term exposure to fine particulate matter and incidence of diabetes in the Danish Nurse Cohort. Environ Int. 2016 May 1;91:243–50.

77. Kr ämer U, Herder C, Sugiri D, Strassburger K, Schikowski T, Ranft U, et al. Traffic-Related Air Pollution and Incident Type 2 Diabetes: Results from the SALIA Cohort Study. Environ Health Perspect. 2010 Sep;118(9):1273–9.

For peer review only



For peer review only - http://bmjopen.bmj.com/site/about/guidelines.xhtml

78. Brook RD, Jerrett M, Brook JR, Bard RL, Finkelstein MM. The relationship between diabetes mellitus and traffic-related air pollution. J Occup Environ Med. 2008 Jan;50(1):32–8.

79. Clougherty JE. A Growing Role for Gender Analysis in Air Pollution Epidemiology. Environ Health Perspect. 2010 Feb;118(2):167–76.

80. Kanner RE, Connett JE, Altose MD, Buist AS, Lee WW, Tashkin DP, et al. Gender difference in airway hyperresponsiveness in smokers with mild COPD. The Lung Health Study. Am J Respir Crit Care Med. 1994 Oct;150(4):956–61.

81. Bennett WD, Zeman KL, Kim C. Variability of fine particle deposition in healthy adults: effect of age and gender. Am J Respir Crit Care Med. 1996 May;153(5):1641–7.

82. Wen M, Zhang X. Contextual Effects of Built and Social Environments of Urban Neighborhoods on Exercise: A Multilevel Study in Chicago. Am J Health Promot. 2009 Mar 1;23(4):247–54.

83. Fitzmaurice GM, Ravichandran C. A primer in longitudinal data analysis. Circulation. 2008 Nov 4;118(19):2005–10.

84. Stephens C. The urban environment, poverty and health in developing countries. Health Policy Plan. 1995 Jun;10(2):109–21.

85. Urbanization and Migration in Developing Asia [Internet]. 2022 [cited 2022 Nov 24]. Available from: https://thediplomat.com/2015/09/urbanization-and-migration-in-developing-asia/

86. Miller ST, Schlundt DG, Pichert JW, Ahmed NU. Shaping Environments for Reductions in Type 2 Diabetes Risk Behaviors: A Look at CVD and Cancer Interventions. Diabetes Spectr. 2002 Jul 1;15(3):176–82.

For peer review only



For peer review only - http://bmjopen.bmj.com/site/about/guidelines.xhtml

For peer review only



For peer review only - http://bmjopen.bmj.com/site/about/guidelines.xhtml

**Fig 1. Study identification, screening, and eligibility, guided by PRISMA for T2D outcome.**

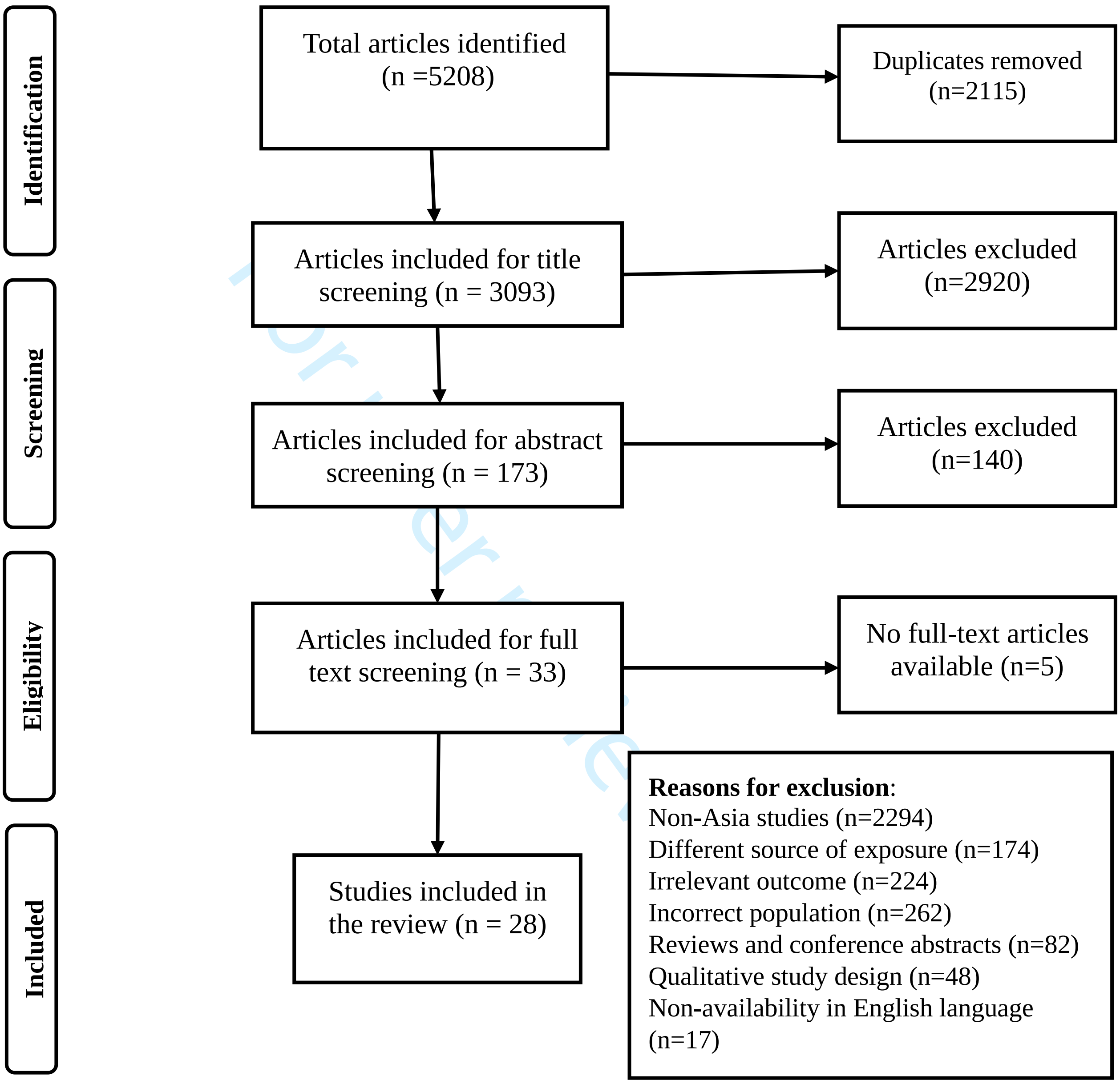

**Online Supplement-1**

**Systematic literature search strategy in Medline, Embase and Global Health using OVID platform**

| Concepts | No. | Term |
|---|---|---|
| Built Environment | 1 | (environment$ adj1 (design$ or green$ or pollut$ or built or food or natur$ or determinant$ or influence or support$ or approach$ or variable$ or attribute$ or barrier$ or local$ or rural$ or urban$ or object$ or perceiv$ or measure$ or obes$ or residen$ characteris$ or correlate$ or soci$ or physical$)).mp. |
| | 2 | (travel$ adj1 (active$ or passive$)).tw. |
| | 3 | (plan$ adj1 (urban$ or town$ or city$)).tw. |
| | 4 | (park$ adj1 (access$ or urban$)).tw. |
| | 5 | (space$ adj1 (green$ or open$ or natur$)).tw. |
| | 6 | (fast food$ adj1 (dens$ or restaurant$)).tw. |
| | 7 | (food$ adj1 (outlet$ or suppl$)).tw. |
| | 8 | (facilit$ adj1 (recreation$ or sport$)).tw. |
| | 9 | (pollut$ adj1 (air$ or noise$)).tw. |
| | 10 | (residen$ adj1 (loca$ or proximit$)).tw. |
| | 11 | retail densit*.tw. |
| | 12 | walkability.mp. |
| | 13 | contextual research.tw. |
| | 14 | sprawl$.ab. |
| | 15 | zoning.ab. |
| | 16 | worksite*.ab. |
| | 17 | cyclability.ab. |
| | 18 | sidewalk$.ab. |
| | 19 | crowd$.ab. |
| | 20 | pedestrian$.ab. |
| | 21 | cyclepath$.ab. |
| | 22 | aesthetic*.ab. |
| | 23 | remote$.ab. |

For peer review only

| | | |
|---|---|---|
| | 24 | neighbo?rhood*.mp. |
| | 25 | 2 or 3 or 4 or 5 or 6 or 7 or 8 or 9 or 10 or 11 or 12 or 13 or 14 or 15 or 16 or 17 or 18 or 19 or 20 or 21 or 22 or 23 or 24 |
| Type 2 T2D mellitus | 26 | hyperglyc$.mp. |
| | 27 | T2D.ab. |
| | 28 | glyc?emi$.mp. |
| | 29 | (glucose adj1 (blood or high or low or $tolerance)).tw. |
| | 30 | insulin resistan$.tw. |
| | 31 | hba1$.mp. |
| | 32 | hyp?glyc$.mp. |
| | 33 | 26 or 27 or 28 or 29 or 30 or 31 or 32 |
| Observational studies | 34 | epidem$.mp. |
| | 35 | disease association.tw. |
| | 36 | inciden$.mp. |
| | 37 | prevalen$.mp. |
| | 38 | 34 or 35 or 36 or 37 |
| Asia | 39 | asia$.mp. |
| | 40 | (Afghanistan$ or Armenia$ or Azerbaijan or Bahrain or Bangladesh$ or Bhutan$ or Brunei$ or Cambodia$ or Chin$ or Cyprus or Georgia$ or India$ or Indonesia$ or Iran$ or Iraq$ or Israel$ or Japan$ or Jordan or Kazakhstan or Kuwait or Kyrgyzstan or Laos or Leban$ or Malaysia$ or Maldives or Mongolia$ or Myanmar or Burm$ or Nepal$ or North Korea$ or Oman or Pakistan$ or Palestin$ or Philippines or Qatar$ or Russia$ or Saudi Arabia$ or Singapor$ or South Korea$ or Sri Lanka$ or Syria$ or Taiwan or Tajikistan or Thailand or Timor-Leste or Turk$ or Turkmenistan or United Arab Emirates or Uzbekistan or Vietnam$ or Yemen).mp. |
| | 41 | 39 or 40 |
| Complete Search | 42 | 1 and 25 and 33 and 38 and 41 |

For peer review only

## Online Supplement-2

### Key to Quality Assessment of the selected studies

| Criteria | Items | Description | Maximum Score |
|---|---|---|---|
| **Selection** | **Representativeness of the sample** | The studies choose the samples which were truly or somewhat representative of the average in the target population or not. | 1 |
| | **Study size** | The sample size the study selected was justified and satisfactory or not | 2 |
| | **Ascertainment of the exposure** | If the study applied validated measurement tool to ascertain the risk factors, we assigned two stars. Additionally, we still assigned one star to the study applied non-validated measurement tool which was available or described. | 2 |
| **Comparability** | **The Subjects in Different Outcome Groups are Comparable** | The study controlled for the confounding factors or not | 1 |
| **Outcome measures and analysis** | **Assessment of the outcome** | The study applied independent blind assessment, record linkage, self-report or no description. If it used independent blind assessment or record linkage, we assigned two stars. If it used self-report, we assigned one star. | 2 |
| | **Statistical test** | If the statistical test used to analyze the data was clearly described and appropriate, and the measurement of the association was presented, including confidence intervals and the probability level (i.e., p value), we assigned one star. | 1 |

Based on standard reference Newcastle-Ottawa Quality Assessment scale(15)



For peer review only - http://bmjopen.bmj.com/site/about/guidelines.xhtml

**Quality score for eligible studies**

| S.No | Source | Selection | | | Comparability | Outcome measures and analysis | | Total (9) | Quality |
|---|---|---|---|---|---|---|---|---|---|
| | | Representativeness of the sample | Study size | Ascertainment of the exposure | The subjects in different outcome groups are comparable | Assessment of the outcome | Statistical test | | |
| 1. | Chuang et al 2011(34) | 1 | 2 | 2 | 1 | 2 | 0 | 8 | Very good |
| 2. | Tong et al 2015 (45) | 1 | 2 | 2 | 1 | 2 | 1 | 9 | Very good |
| 3. | Lee et al 2015 (30) | 1 | 2 | 2 | 1 | 2 | 0 | 8 | Very good |
| 4. | Chen et al 2016 (43) | 1 | 2 | 2 | 1 | 2 | 1 | 9 | Very good |
| 5. | Liu Fei et al 2016 (35) | 1 | 2 | 2 | 1 | 2 | 0 | 8 | Very good |
| 6. | Zhang et al 2017 (22) | 1 | 2 | 1 | 1 | 2 | 0 | 7 | Good |
| 7. | Fujiwara et al 2017(29) | 1 | 2 | 2 | 1 | 2 | 0 | 8 | Very good |
| 8. | Biswas and Kabir 2017 (32) | 1 | 2 | 2 | 1 | 2 | 1 | 9 | Very good |
| 9. | Khafaie et al 2017 (41) | 1 | 2 | 2 | 1 | 2 | 0 | 8 | Very good |
| 10. | Sohn and Oh 2017(42) | 1 | 2 | 2 | 1 | 2 | 1 | 9 | Very good |
| 11. | Qiu et al 2018 (39) | 1 | 2 | 2 | 1 | 2 | 1 | 9 | Very good |
| 12. | Liu et al 2019 (47) | 1 | 2 | 2 | 1 | 2 | 1 | 9 | Very good |



For peer review only - http://bmjopen.bmj.com/site/about/guidelines.xhtml

| | | | | | | | | | |
|---|---|---|---|---|---|---|---|---|---|
| 13. | Liang et al 2019 (36) | 1 | 2 | 2 | 1 | 2 | 0 | 8 | Very good |
| 14. | Curto et al 2019 (23) | 1 | 2 | 2 | 1 | 2 | 0 | 8 | Very good |
| 15. | Han et al 2019 (40) | 1 | 1 | 2 | 1 | 2 | 1 | 8 | Very good |
| 16. | Lin et al 2019(26) | 1 | 2 | 2 | 1 | 2 | 1 | 9 | Very good |
| 17. | Yang et al 2019 (25) | 1 | 2 | 2 | 1 | 1 | 1 | 8 | Very good |
| 18. | Jacob et al 2019 (33) | 0 | 1 | 2 | 0 | 2 | 0 | 5 | Satisfactory |
| 19. | Li et al 2019 (31) | 1 | 1 | 2 | 1 | 2 | 1 | 9 | Very good |
| 20. | Tao et al 2019 (37) | 1 | 2 | 2 | 1 | 2 | 1 | 9 | Very good |
| 21. | Yang et al 2019(27) | 1 | 2 | 2 | 1 | 2 | 1 | 9 | Very good |
| 22. | Leng et al 2020 (28) | 1 | 2 | 1 | 1 | 2 | 1 | 8 | Very good |
| 23. | Wong et al 2020 (46) | 1 | 2 | 2 | 1 | 2 | 1 | 9 | Very good |
| 24. | Wang et al 2020 (24) | 1 | 2 | 2 | 1 | 2 | 1 | 9 | Very good |
| 25. | Mehdinia et al 2020 (38) | 1 | 2 | 2 | 1 | 2 | 1 | 9 | Very good |
| 26. | Shan et al 2020 (44) | 1 | 2 | 1 | 1 | 2 | 1 | 8 | Good |



For peer review only - http://bmjopen.bmj.com/site/about/guidelines.xhtml

**Online Supplement -3**

**Summary of study characteristics of articles**

| **Author, year & country** | **Study design** | **Sample size & Age (in years)** | **Outcome type** | **Exposure category** | **Exposure assessment** | **Confounders** | **Statistical method** | **Study Findings** |
|---|---|---|---|---|---|---|---|---|
| Chuang et al, 2011, Taiwan (41) | Cross-sectional | 1023 participants aged ≥ 54 years | T2D risk | Air pollution | Secondary | Age, sex, body mass index, current smoking, drinking, and smooth functions of visit date and yearly temperature | Linear additive models | There was a stronger association between T2D and PM2.5 than with two gases, O3 and NO2. |
| Tong et al, 2015, China(53) | Cross-sectional | 1430 participants from all age group | T2D risk | Air pollution | Secondary | Seasonality, temperature, humidity | Log linear generalized model | Increase in 2-day average concentrations of particulate matters like (PM10), SO2, and NO2 correspond to increases in T2D morbidity. The association was in expected direction |
| Lee et al, 2015, South Korea(36) | Cross-sectional | 16178 participants aged ≥20 years | T2D prevalence | Walkability | Secondary | Age, sex, smoking status, drinking status and income level | Multiple logistic regression analysis | T2D mellitus is associated with walkable environments, and physical activity may contribute to the association. |

For peer review only

| | | | | | | | | |
|---|---|---|---|---|---|---|---|---|
| Chen et al, 2016, China(51) | Cohort | 27685 participants aged between18-98 years | T2D prevalence | Air pollution | Secondary | Age, sex, blood pressure (systolic/diastolic blood pressure),bmi, diabetes, treatment of diabetes, exercise activity, walking, smoking, alcohol drinking, health examination hospital, seasonality, temperature and relative humidity | Longitudinal analysis | In women, elderly and overweight people, air pollutants had a higher effect on FBG than in males, young and underweight people. This suggests that air pollution may increase the levels of FBG. |
| Liu et al, 2016, China(42) | Cross-sectional | 11847 participants aged ≥45 years | T2D prevalence and risk | Air pollution | GIS | Location of residence (urban or rural), age, sex, educational level (low, medium, high), body mass index, smoking status (current smoker, ex- smoker and non-smoker), pack years for current smokers, drinking (>1/month, <1/month, never), type of heating energy: central heating, clean, unclean, other; type of cooking energy: clean, unclean, other) and ozone (estimated by the gbd project) | Multivariable logistic regression | Significant increases in T2D prevalence, fasting glucose levels, and hba1c levels were associated with long-term exposure to PM2.5. |

For peer review only

| | | | | | | | | |
|---|---|---|---|---|---|---|---|---|
| Zhang et al, 2017, China (28) | Cross-sectional | 2745 participants aged ≥18 years | T2D Prevalence | Accessibility to services | Secondary | County- level socioecono mic factors (e.g., natural logarithms of the local gdp per capita and the local averaged wage), health facility factors (e.g.,hospital density and health center density), demographi c features (e.g., household size, regional average age and county-level gender divisions) and other health outcomes probably influencing diabetes (e.g., obesity and hypertensio n) | Ordinary least squares, robust regression and a set of binary choice model | China's T2D prevalence is inextricably linked to the built environment as well as individual economic factors. When combined with other influential factors, China's strong economic growth has made obesity and T2D more common. |
| Fujiwara et al, 2017, Japan (35) | Cross-sectional | 8904 participants aged ≥ 65 years | T2D Prevalence | Food environment and walkability | GIS | Each hilly assessment, perception of access to grocery stores (unit: iqr), number of parks (unit: iqr), number of hospitals (unit: iqr), population density (tertile), and land value (unit: iqr), sex, marital status, household number, income, working status, drinking, smoking, vegetable consumption, walking, going-out behavior, frequency of meeting, bmi (category), and depression | Multilevel logistic regression | There was no association between hilly neighbourhoods and T2D mellitus, but they protected against poorly managed T2D mellitus. |

For peer review only

| | | | | | | | | |
|---|---|---|---|---|---|---|---|---|
| Biswas and Kabir, 2017, Bangladesh (39) | Cross-sectional | 7544 participants aged ≥35 years | T2D Prevalence | Availability and accessibility to services | Secondary | | Generalized linear mixed models | It was found that T2D was not significantly associated with distance from a health facility. |
| Khafaie et al, 2017, India(49) | Cross-sectional | 1213 participants aged ≥46 years | T2D risk | Air pollution | Atmospheric dispersion model | Age, gender, duration of diabetes, WHR, smoking and season of enrolment | Multiple linear regression | Exposure to PM10 long-term correlates with high insulin resistance and high glycaemia. |
| Sohn and Oh, 2017, South Korea(50) | Cross-sectional | 43941 participants with median age 46.7 | T2D prevalence | Air pollution | Secondary | Age, body mass index, economic activity status, educational attainment, and smoking behavior | Multivariable logistic regression | In South Korea, PM10 and SO2 levels may be associated with T2D prevalence, but there appears to be a gender difference. Exposures to either PM10 or SO2 were significantly related to the prevalence of DM 2 among women, but not among men. |
| Qiu et al, 2018, China(46) | Cohort | 53905 participants aged ≥65 years | T2D incidence and prevalence | Air pollution | GIS | Age, sex, calender year of entry, bmi, smoking status, alcohol drinking, physical exercise, education, monthly expenses, medication taken, self reported comorbidity including hypertension, heart disease, COPD/asthma, or cerebrovascular accident at baseline, small area characteristics (% 65+ years, % with<br>tertiary education and income ≥ US$ 1923/month) and district | Cox regression | In Hong Kong's elderly population, long-term exposure to high levels of PM2.5 may increase the prevalence and incidence of type 2 T2D mellitus. |

For peer review only

| | | | | | | | | |
|---|---|---|---|---|---|---|---|---|
| | | | | | | smoking rate. | | |
| Liu et al, 2019, China(55) | Cohort | 39191 participants aged between 18-79 years | T2D prevalence and risk | Air pollution | GIS | Age, sex, education level, marital status, average monthly income, smoking, drinking, high fat diet, fruit and vegetable intake, physical activity, family history of diabetes, bmi | Linear regression model | There is a positive correlation between higher exposures of air pollutants and increased odds of developing type 2 T2D, along with higher fasting blood sugar levels among Chinese rural residents. |

For peer review only

| | | | | | | | | |
|---|---|---|---|---|---|---|---|---|
| Liang et al, 2019, China(43) | Cohort | 88397 participants with mean age 51.7 | T2D incidence | Air pollution | GIS | Age, gender, temperature, RH, BMI, smoking status, educational level, work- related physical activity level, family history of diabetes and hypertension status, neighborhood SES including urbanicity (urban or rural) and county-level averaged years of education | Cox proportional hazard regression | There is evidence that prolonged exposure to PM2.5 is associated with T2D incidence in China. If air quality is improved in China, it will impact the reduction of T2D incidence in China. |
| Curto et al, 2019, India (29) | Cross-sectional | 5065 participants aged ≥18 years | T2D Prevalence | Air pollution | Gravimetric sampler | Pm2.5/bc residual + age + sex + mean pm2.5/bc village + (1\| village/ household) + sugar and sweets intake + physical activity + education + alcohol intake + smoking + environmental tobacco smoke + standard of living index + cooking fuel + body mass index + waist-to-hip-ratio + physician- diagnosed hypertension | Logistic mixed regression models | Women's blood glucose levels were negatively influenced by PM2.5 and black carbon (BC). In men, associations were negative for PM2.5 and positive for BC. |
| Han et al, 2019, China(47) | Case-control | 120 participants in each group aged between 50-65 | T2D prevalence | Air pollution | Particulate sampler | Age, sex, BMI, smoking status, medication usage, disease history, day of the week (DOW), temperature and RH | Linear mixed effect model | People with T2D are more susceptible to the acute cardio metabolic effects of air pollution than healthy people. |

| | | | | | | | | |
|---|---|---|---|---|---|---|---|---|
| | | years | | | | | | |
| Lin et al, 2019, Taiwan (32) | Cross-sectional | 341211773602 participants aged ≥ 18 years | T2D risk | Urban green space | GIS | - | Linear mixed-effect model with an individual-level random effect | Residential greenness appears to be associated with lower glucose levels. Physical activity is a possible mediator of this association. |
| Yang et al, 2018, China(31) | Cross-sectional | 15477 participants aged ≥ 50 years | T2D prevalence | Air pollution | Secondary | Age, sex, body-mass index, education, family income, smoking, alcohol consumption, exercise, low calorie and low-fat controlled diet, sugar-sweetened soft drink consumption, family history of diabetes, and district | Linear regression model | T2D and PM2.5 are statistically significantly associated. In stratified analyses by fruit consumption, a lower association was found among participants. |
| Jacob et al, 2019, India (40) | Cross-sectional | 410 participants aged ≥18 years | T2D prevalence | Air pollution | Gravimetric sampler | | Multivariable logistic regression | People living in areas with high particulate matter exposure were 77.5% more likely to have T2D than those living in areas with less exposure. |

For peer review only

| | | | | | | | | |
|---|---|---|---|---|---|---|---|---|
| Li et al, 2019, India(37) | Cross-sectional | 5764 participants with median age 28.8 | T2D risk | Food environment | GIS | Age, sex, education, occupation, standard of living index, tobacco, alcohol, and physical activity + densities or distances of highly processed/take-away food vendors | Three-level mixed-effects linear regression | Having fewer fruit and vegetable vendors and having more take-out and highly processed foods was associated with higher cardiovascular risk profiles. |
| Tao et al, 2019, China(44) | Cross-sectional | 6627 participants aged ≥18 years | T2D risk | Air pollution | GIS | Sex, age, race, education, occupation, household income, marital status, type of home cooking fuel, family history of diabetes mellitus, smoking, passive smoking, alcohol consumption, BMI, weight change in the past year, blood lipid control, blood pressure control, season of investigation, physical activity, weekly consumption of grains, vegetables, fruit, meat products and Pm1 exposure | Multinomial logistic regression | PM2.5 and PM1 were both linked to an increased risk of impaired fasting glucose (IFG) and T2D. |

For peer review only

| | | | | | | | | |
|---|---|---|---|---|---|---|---|---|
| Yang et al., 2019, China(33) | Cross-sectional | 15,477 participants aged 18-74 years | T2D Prevalence | Urban green space | GIS | Age (years), sex (male vs. female), ethnicity (Han vs. other), household annual income (≤5000 Yuan, 5001-10,000 Yuan, 10,001-30,000 Yuan, ≥ 30,000 Yuan), and highest educational attainment (no school, primary school, middle school, junior college or higher). Air pollution, physical activity and BMI were also selected as candidate mediators | Linear regression model | Higher residential greenness appears to be beneficially associated with both T2D prevalence and glucose-homeostasis markers in Chinese adults. |
| Leng et al, 2020, China(34) | Cross-sectional | 4155 participants aged ≥20 years | T2D prevalence | Urban green space | Secondary | Age, gender, education | Logistic regression | Green space characteristics of a neighborhood were not associated with T2D. |
| Wong et al, 2020, Malaysia (54) | Cross-sectional | 56710 participants from all age group | T2D risk | Air pollution | Secondary | Age, gender, ethnicity, urban/rural areas, body mass index (BMI) and physical activity | Multiple logistic and linear regression model | In Malaysia, long-term exposure to O3 is a significant factor associated with under diagnosed DM. The risk of DM may be influenced by the presence of PM10, NO2 and NOx, and their roles should be explored in further detail with other interaction models. |

For peer review only

| | | | | | | | | |
|---|---|---|---|---|---|---|---|---|
| Wang et al, 2020, China(30) | Cohort | 19884 participants aged between 20-79 | T2D incidence | Air pollution | Secondary | Age, gender, pollution monitoring stations, marital status, educational level, family income, work type, smoking and drinking status, physical exercise, family history of diabetes, BMI, dietary salt, oil, sugar intake, average temperature, relative humidity, the season at baseline, hypertension status, TC, TG, LDLC and HDLC | Cox proportional hazard regression | T2D incidence increased by 17% per 10μg/m3 increase in environmental PM10, and the risk rises gradually with higher PM10 concentrations. The risk of T2D incidence associated with ambient PM10 was higher among females, young to middle-aged people, overweight and obese subjects, and those with a FPG level at baseline less than 5.6 mmol/L. |
| Mehdinia et al, 2020, Iran(45) | Cross-sectional | 482833 participants aged between 30-59 years | T2D prevalence | Air pollution | Secondary | | Linear regression model | T2D prevalence and PM2.5 concentrations show a significant association. |
| Shan et al, 2020, China(52) | Cohort | 38529 participants with mean age 44.1 | T2D incidence | Air pollution | Secondary | Sex, age, smoking status, education level, personal income, marital status, occupational exposure, exercise, fruit consumption, vegetable consumption, seafood consumption, meat consumption, poultry consumption and alcohol consumption | Cox proportional hazard regression | Among Chinese adults, it was found that long-term exposure to high levels of PM10, SO2, and NO2 increased risk of T2DM incidence and mortality. |

For peer review only

| | | | | | | | | |
|---|---|---|---|---|---|---|---|---|
| Kusuma et al, 2022, South Asia(38) | Cross sectional | 12167 participants with median age 45.5 | T2D prevalance | Food environment | GIS | Family history of diabetes, BMI, dietary salt, oil, sugar intake, average temperature, relative humidity, the season at baseline, hypertension status, TC, TG, LDLC and HDLC | Ordinary least squares multivariate regression | An increase in blood glucose levels and the likelihood of being diagnosed with diabetes is associated with having at least one FFR locally by 16% and 19% respectively. FFR density had a stronger positive association with blood glucose levels among women than men. In contrast, FFR proximity had a stronger association with blood glucose levels among men and those with higher incomes. |
| Liu et al, 2023, China(48) | Cohort | 19121 participants aged ≥45 years | T2D risk | Air pollution | Secondary | Demographic characteristics, indoor pollution, life behaviours and ambient temperature | Cox proportional hazards regression model | The long-term exposure to PM2.5 can increase the risk of type 2 diabetes and make them more prone to arthritic problems. |

# PRISMA2020Checklist

| Section and Topic | Item # | Checklist item | Location where item is reported |
|---|---|---|---|
| **TITLE** | | | |
| Title | 1 | Identify the report as a systematic review. | Page 1 |
| **ABSTRACT** | | | |
| Abstract | 2 | See the PRISMA 2020for Abstracts checklist. | Page 2 |
| **INTRODUCTION** | | | |
| Rationale | 3 | Describe the rationale for the review in the context of existing knowledge. | Page 4 |
| Objectives | 4 | Provide an explicit statement of the objective(s) or question(s) the review addresses. | Page 5 |
| **METHODS** | | | |
| Eligibility criteria | 5 | Specify the inclusion and exclusion criteria for the review and how studies were grouped for the syntheses. | Page 5, Table 1 |
| Information sources | 6 | Specify all databases, registers, websites, organizations, reference lists and other sources searched or consulted to identify studies. Specify the date when each source was last searched or consulted. | Page 7 |
| Search strategy | 7 | Present the full search strategies for all databases, registers and websites, including any filters and limits used. | Online supplement File 1 |
| Selection process | 8 | Specify the methods used to decide whether a study met the inclusion criteria of the review, including how many reviewers screened each record and each report retrieved, whether they worked independently, and if applicable, details of automation tools used in the process. | Page 6 |
| Data collection process | 9 | Specify the methods used to collect data from reports, including how many reviewers collected data from each report, whether they worked independently, any processes for obtaining or confirming data from study investigators, and if applicable, details of automation tools used in the process. | Page 7-8 |
| Data items | 10a | List and define all outcomes for which data were sought. Specify whether all results that were compatible with each outcome domain in each study were sought(e.g. for all measures, time points, analyses),and if not, the methods used to decide which results to collect. | Online supplement File 3 |
| | 10b | List and define all other variables for which data were sought(e.g. participant and intervention characteristics ,funding sources).Describe any assumptions made about any missing or unclear information. | Page 7-8 |
| Study risk of bias assessment | 11 | Specify the methods used to assess risk of bias in the included studies, including details of the tool(s) used, how many reviewers assessed each study and whether they worked independently, and if applicable, details of automation tools used in the process. | Page 7 |
| Effect measures | 12 | Specify for each outcome the effect measure(s)(e.g. risk ratio, mean difference) used in the synthesis or presentation of results. | Online supplement File 3 |
| Synthesis methods | 13a | Describe the processes used to decide which studies were eligible for each synthesis(e.g. tabulating the study intervention characteristics and comparing against the planned groups for each synthesis(item#5)). | Page 8-9, Online supplement file 2 |
| | 13b | Describe any methods required to prepare the data for presentation or synthesis, such as handling of missing summary statistics, or data conversions. | NA |

For peer review only - http://bmjopen.bmj.com/site/about/guidelines.xhtml

PRISMA 2020 Checklist

| | | | |
|---|---|---|---|
| | 13c | Describe any methods used to tabulate or visually display results of individual studies and syntheses. | Online supplement file 3 |
| | 13d | Describe any methods used to synthesize results and provide a rationale for the choice(s). If meta-analysis was performed, describe the model(s), method(s) to identify the presence and extent of statistical heterogeneity, and software package(s) used. | Page 8-9 |
| | 13e | Describe any methods used to explore possible causes of heterogeneity among study results (e.g. subgroup analysis, meta-regression). | NA |
| | 13f | Describe any sensitivity analyses conducted to assess robustness of the synthesized results. | NA |
| Reporting bias assessment | 14 | Describe any methods used to assess risk of bias due to missing results in a synthesis (arising from reporting biases). | NA |
| Certainty assessment | 15 | Describe any methods used to assess certainty (or confidence) in the body of evidence for an outcome. | NA |

For peer review only

# PRISMA2020Checklist

| Sectionand Topic | Item # | Checklist item | Location whereitem isreported |
|---|---|---|---|
| **RESULTS** | | | |
| Studyselection | 16a | Describetheresults ofthesearchandselection process,from thenumberofrecordsidentifiedin thesearch tothenumber ofstudiesincludedin thereview,ideallyusingaflowdiagram. | Page 11-12, Figure 1 |
| | 16b | Cite studiesthatmightappearto meettheinclusioncriteria,butwhichwereexcluded, andexplainwhy theywereexcluded. | NA |
| Study characteristics | 17 | Cite eachincludedstudyandpresent itscharacteristics. | Online supplement file 3 |
| Riskofbias in studies | 18 | Present assessmentsofriskofbias foreach includedstudy. | NA |
| Resultsof individualstudies | 19 | For all outcomes, present, foreachstudy:(a)summary statisticsforeach group(whereappropriate) and(b)aneffectestimateanditsprecision (e.g.confidence/credibleinterval),ideallyusingstructured tablesor plots. | Online supplement file 3 |
| Resultsof syntheses | 20a | For eachsynthesis, brieflysummarisethecharacteristicsandrisk ofbiasamongcontributing studies. | NA |
| | 20b | Presentresults ofallstatisticalsynthesesconducted.Ifmeta-analysiswasdone,presentforeach thesummaryestimateand its precision(e.g. confidence/credible interval) andmeasuresofstatistical heterogeneity.Ifcomparinggroups, describe the directionoftheeffect. | Page 16-17 |
| | 20c | Presentresults ofallinvestigationsofpossiblecausesofheterogeneityamong studyresults. | NA |
| | 20d | Presentresults ofallsensitivity analysesconducted toassess therobustness ofthesynthesizedresults. | NA |
| Reportingbiases | 21 | Present assessmentsofriskofbias due tomissingresults(arising fromreportingbiases)foreachsynthesisassessed. | NA |
| Certainty of evidence | 22 | Present assessmentsofcertainty(orconfidence) in thebodyofevidence foreach outcomeassessed. | NA |
| **DISCUSSION** | | | |
| Discussion | 23a | Provideageneralinterpretation oftheresults in thecontextof otherevidence. | Page 17-19 |
| | 23b | Discussanylimitationsoftheevidenceincluded inthereview. | Page 20 |
| | 23c | Discussanylimitationsofthereviewprocesses used. | Page 20 |
| | 23d | Discussimplications oftheresults for practice, policy,andfutureresearch. | Page 20-21 |
| **OTHER INFORMATION** | | | |
| Registrationand protocol | 24a | Provideregistrationinformation for thereview,includingregister name andregistration number,or statethatthereviewwas notregistered. | Page 6 |
| | 24b | Indicatewherethereview protocolcan beaccessed, orstatethataprotocolwas notprepared. | Page 6 |
| | 24c | Describeandexplain anyamendments toinformationprovided atregistrationor in theprotocol. | NA |
| Support | 25 | Describesources offinancialor non-financialsupportforthereview,andtheroleofthefunders orsponsorsin thereview. | Page 21 |
| Competing interests | 26 | Declare anycompetinginterestsofreview authors. | NA |
| Availability of data,code and othermaterials | 27 | Reportwhichofthe followingare publiclyavailableandwhere theycanbe found:templatedatacollectionforms;dataextracted from included studies; datausedfor allanalyses;analyticcode;anyothermaterialsusedinthereview. | NA |

For peer review only - http://bmjopen.bmj.com/site/about/guidelines.xhtml

*From:* PageMJ,McKenzieJE,BossuytPM,BoutronI,HoffmannTC,MulrowCD,etal.ThePRISMA2020statement:anupdatedguidelineforreportingsystematicreviews.BMJ2021;372:n71.doi:

10.1136/bmj.n71

# PRISMA2020Checklist

For moreinformation,visit: http://www.prisma-statement.org/

For peer review only
